# Chloroplast genomes of *Eriobotrya elliptica* and an unknown wild loquat “YN-1”

**DOI:** 10.1038/s41598-024-69882-7

**Published:** 2024-08-13

**Authors:** Zhicong Lin, Qing Guo, Shiwei Ma, Hailan Lin, Shunquan Lin, Shoukai Lin, Jincheng Wu

**Affiliations:** 1 College of Environmental and Biological Engineering, Fujian Provincial Key Laboratory of Ecology-Toxicological Effects and Control for Emerging Contaminants, Key Laboratory of Ecological Environment and Information Atlas (Putian University) Fujian Provincial University, Putian University, Putian, 351100 China; 2 College of Environmental and Biological Engineering, Putian University, Putian, 351100 China

**Keywords:** Chloroplast genome, Wild loquat, RNA editing, Phylogeny, Evolution, Genetics

## Abstract

The chloroplast genomes of wild loquat can help to determine their place in the history of evolution. Here, we sequenced and assembled two novel wild loquat’s chloroplast genomes, one is *Eriobotrya elliptica*, and the other is an unidentified wild loquat, which we named “YN-1”. Their sizes are 159,471 bp and 159,399 bp, respectively. We also assembled a cultivated loquat named ‘JFZ’, its chloroplast genome size is 159,156 bp. A comparative study was conducted with six distinct species of loquats, including five wild loquats and one cultivated loquat. The results showed that both *E. elliptica and* “YN-1” have 127 genes, one gene more than *E. fragrans,* which is *psbK*. Regions *trnF-GAA*-*ndhJ, petG*-*trnP-UGG*, and *rpl32*-*trnL-UAG* were found to exhibit high variability. It was discovered that there was a positive selection on *rpl22* and *rps12*. RNA editing analysis found several chilling stress-specific RNA editing sites, especially in *rpl2* gene. Phylogenetic analysis results showed that “YN-1” is closely related to *E. elliptica*, *E. obovata* and *E. henryi*.

## Introduction

The chloroplast genomes always contain two inverted repeats (IR), one large single copy (LSC), and one small single copy (SSC)^1^. The SSC can exhibit reverse complementary in different haplotypes of some chloroplast genomes^2^. Chloroplast genome sequences are useful markers for species identification^3^, whether using the entire chloroplast genome or specific fragments such as simple sequence repeats (SSR) or variable regions. Plant phylogenetic classification can also be studied using boundaries that vary among LSC, SSC, and IRs^4^. Most chloroplast genomes contain 110 to 130 genes, including protein-coding genes, rRNAs, and tRNA genes^5^. These genes are involved in fatty acid and amino acid synthesis pathways, as well as in the process of photosynthesis^4,6^. RNA editing of genes in the chloroplast genome is a post-transcriptional modification process, and these have been reported in some plants^7–9^. Cytidine to uridine conversion RNA editing events occur frequently, and occasionally an adenosine to inosine transition takes place^8^. The C-to-U editing rates were reported to be significantly reduced under heat or cold stress compared to normal conditions^10^.

*Eriobotrya japonica* (Thunb.) Lindl. (cultivated loquat, also known as common loquat) is a perennial fruit tree in the Rosaceae family, similar to apple and peach^11,12^. It is commonly grown in China and other countries^12^. The fruits of loquats are delicious, and their leaves are also used as a material in Chinese medicine containing triterpene^13,14^. Wild loquats are important materials for breeding programs aimed at cultivating loquats because they may be more resistant to stressful environments or have specific colors in their fruits^15^. Several wild loquats’ chloroplast genomes had been sequenced, like *Eriobotrya malipoensis, Eriobotrya bengalensis* et al.^16–18^, but no comparisons have been made among them.

Here, we sequenced and assembled the chloroplast genomes of two wild loquats, one is *Eriobotrya elliptica*, and the other is an unknown wild loquat (At present, we are calling it “YN-1”) that we found in Yunnan province, China. “YN-1” leaves are oval in shape; however, they are smaller than those of *E. elliptica*, measuring 10–15 cm long and 4.4–7 cm wide. Furthermore, the petiole base of YN-1 has hair, and its leaves are smooth and hairless. To date, the tallest "YN-1" tree we have seen is higher than ten meters in height. We also assembled the chloroplast genome of a cultivated loquat, *Eriobotrya japonica* ‘Jiefangzhong’. The chloroplast genomes of five wild loquats and one cultivated variety, "Jiefangzhong," were compared globally. Phylogenetic evolution analysis was conducted using all the published wild loquats, including the two newly assembled wild loquats and one cultivated loquat (*Eriobotrya japonica* ‘Jiefangzhong’).

## Result

### Chloroplast genomes global comparison

Here, we assembled two novel wild loquat chloroplast genomes, *Eriobotrya elliptica* (TY) and “YN-1” (an unknown wild loquat found in Yunnan Province), and one cultivated loquat chloroplast genome (‘Jiefangzhong’, JFZ). We also utilized three other publicly available wild loquats chloroplast genomes (*Eriobotrya bengalensis*, here named as NY; *Eriobotrya malipoensis*, here named as MLP; *Eriobotrya fragrans*, here named as XH) for a comprehensive comparison of the loquat chloroplast genomes.

**Figure 1:**
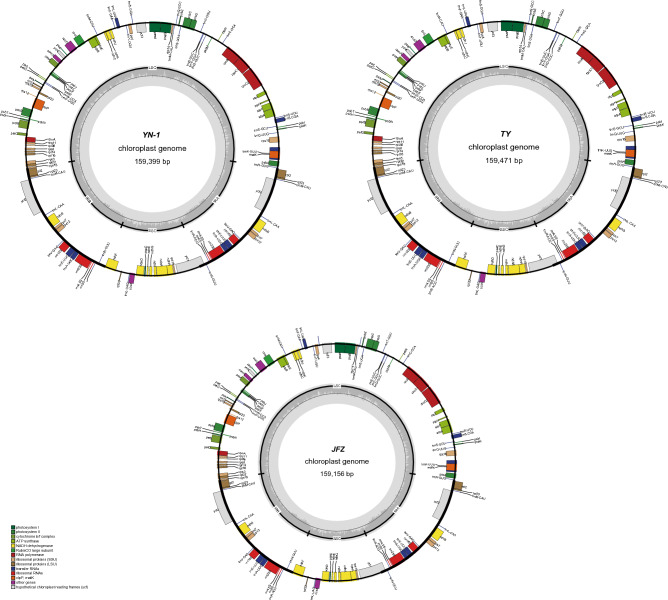
Gene map of the *Eriobotrya elliptica,* “YN-1”, and “JFZ”. Thick lines indicate the extent of the inverted repeat regions (IRa and IRb), which separate the genome into small (SSC) and large (LSC) single copy regions. Genes drawn inside the circle are transcribed clockwise, and those outside are transcribed counter clockwise. Different colors represent different functional groups of genes.

**Figure 2:**
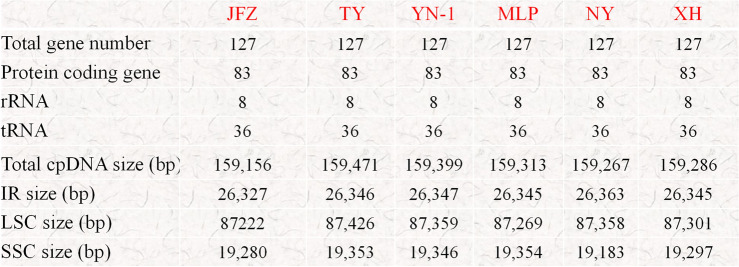
Comparison of gene number, gene type, and different region sizes of of different species of loquats.

**Figure 3:**
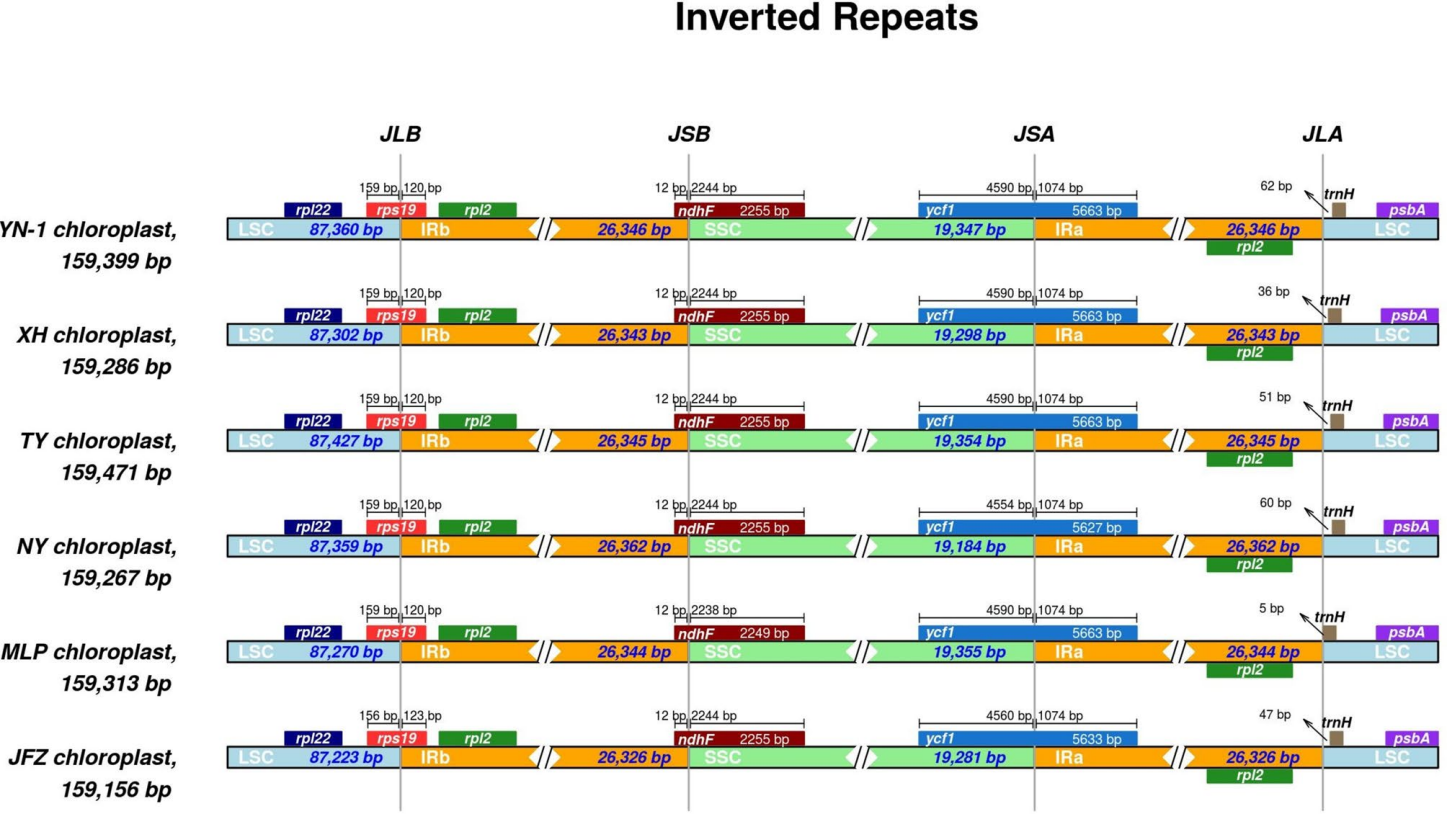
Comparison of the borders of LSC, SSC, and IR regions of chloroplast genomes of different species of loquats.

After assembly, the final chloroplast genome size of *Eriobotrya elliptica* (TY) was 159,471 bp and the chloroplast genome size of the newly discovered wild loquat “YN-1” was 159,399 bp (Fig. 1). The chloroplast genome size of “JFZ” was 159,156 bp (Fig. 1). Although the inverted repeated regions (IRs) of “YN-1” and “TY” only have 1 bp differentiation, the sizes of the large single copy (LSC) and small single copy (SSC) exhibit more differences. The LSC of “TY” was 87,426 bp and the LSC of “YN-1” was 87,359 bp, while the SSC of “TY” was 19,353 bp and 19,346 bp for “YN-1”. Both the chloroplast genomes of “TY” and “YN-1” contain 83 protein-coding genes, 8 rRNA, and 36 tRNA (Fig. 2), similar to the other three kinds of loquats. This is in contrast to the “XH” loquat, which is missing the *psbK* gene. After analyzing the boundaries of the junction sites of the chloroplast genomes, the results showed that there are three genes located in the junction sites: *rps19*, *ndhF*, and *ycf1*. Both *ndhF* in “MLP” and *rps19* in “JFZ” are 3-4 bp different from their corresponding genes in the other four kinds of loquats. Gene *ycf1* in “JFZ” and “NY” showed more variability compared to other loquats, with 30 and 36 bp differences, respectively (Fig. 3). Further alignment analysis revealed that both ycf1 genes in “JFZ” and “NY” contained two identical deletions compared to the *ycf1* gene located in the other four kinds of loquats. Additionally, there was a unique 6 bp deletion “TATCAA”, which is only present in the *ycf1* of “NY” (Fig. S1).

### Structural variation and DNA polymorphism

**Figure 4:**
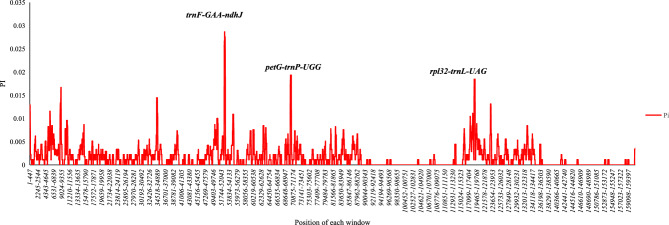
Sliding window analysis (window length: 600 bp, step size: 200 bp) of the whole chloroplast genomes of six different kinds of loquat. X-axis, position of the mid point of a window; Y-axis, nucleotide diversity of each window*.*

We compared the similarity of the six chloroplast genome sequences using mVISTA with the“YN-1” chloroplast genome as a reference. A high degree of synteny exists between the six chloroplast genomes, but the sequences of the CNS are highly variable (Fig. S2). In order to elucidate the sequence nucleotide divergence among six chloroplast genomes, we used DnaSP6.0 to analyze the sequence nucleotide variability within 600 bp window, 200 bp as a step. The highest average sequence divergence is located in *trnF-GAA*-*ndhJ*, followed by *petG*-*trnP-UGG*, and r*pl32*-*trnL-UAG* (Fig. 4). The average DNA sequence variation of different regions differs among various regions, LSC > SSC > IRs (Table S1). We also detected single nucleotide polymorphisms (SNPs) and indels between the two genomes, and we found 717 SNPs and 213 indels (Table S2).

### SSR and repetitive sequences

**Figure 5:**
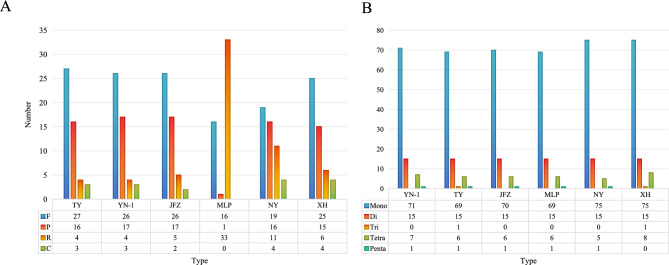
Repeats (**A**) and SSRs (**B**) number comparison of six different species of loquats.

In total, five types of SSRs were detected in the six chloroplast genomes; while only the “TY” and “XH” had tri nucleotide type SSRs. Besides, the penta nucleotide type SSRs were only missed in the “XH” loquat. The number of SSRs detected in different chloroplast genomes ranges from 91 to 99, the “XH” loquat contained the most amount of SSRs (Figure 5B, Table S3). All six chloroplast genomes had 50 repetitive sequences, and only the “MLP” loquat did not have complementary repeats. In terms of the number of repetitive sequences, the “MLP” loquat seems very specific*;* it contained the highest number of inverted repeats while having the fewest palindromic repeats, only 1. The “NY” loquat also had more inverted repeats compared to others, except for the “MLP” loquat. The most popular type of repetitive sequence detected was direct repeats (Fig. 5A). The repeat sizes mainly ranged from 20 to 50 bp, with “YN-1” containing two direct repeats with a size larger than 90 bp, the longest one is 110 bp. The “MLP” loquat chloroplast genome appeared to be very specific; all the repeats’ sizes are larger than 70, with an average size of 78.7 bp (Table S4).

### Codon usage bias

**Figure 6:**
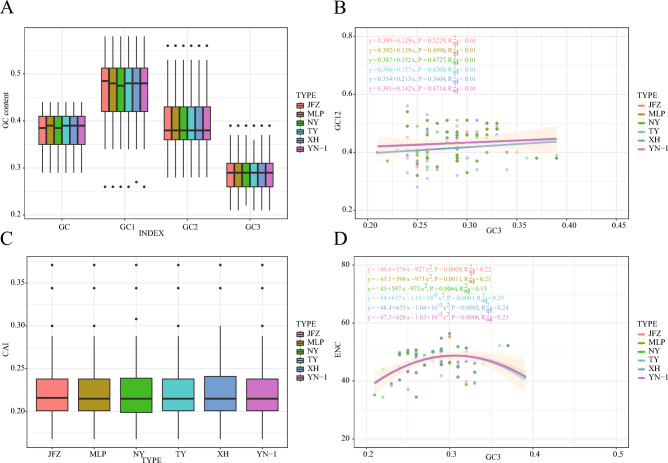
Chloroplast genome codon usage pattern related plot. (**A**) GC content of different codon sites. (**B**) Neutrality plot (GC12 against GC3). (**C**) The codon adaptation index (CAI) value of different function gene sets. (**D**) Relationship between GC3 and effective number of codons (ENC) (ENC-plot).

Protein-coding genes with more than 300 nucleotides were used to explore the codon usage pattern of the six loquat chloroplast genomes^19^. First, we determined each gene’s relative synonymous codon usage (RSCU) value. Across the six varieties of loquats, we observed no discernible differences (Fig S3, Table S5). Then we investigated the base composition of each position of each codon. For the entire gene, the GC content of the “JFZ” and “NY” protein-coding genes is lower than the other four loquats, the median GC content value of “JFZ” and “NY” is 0.385, while for the rest, this value is 0.39 (Fig. 6A). The GC content of the first codon (GC1) in different loquat chloroplast genomes varies. The GC1 median value of the “JFZ” loquat is the highest, which is 0.485; while the GC1 median value of “NY” is the lowest, which is 0.475. The median value of GC1 for the other four kinds of loquat is 0.48. There is no difference in the GC content median value of the second codon (GC2) and the third codon (GC3) among all six kinds of loquat. The correlation between GC12 and GC3 was significant (R2 < 0.01), indicating that mutations affect the CUB in all six types of loquat chloroplast genomes (Fig. 6B). The codon adaptation index (CAI) indicates directional synonymous codon usage bias^20^, only a minor difference between the CAI values of all the six kinds of loquat. The CAI value of “JFZ” is 0.216, while the CAI value for all the remaining varieties is 0.215 (Fig. 6C). The effective number of codons (ENC) value is used to quantify how far the codon usage of a gene departs from the equal usage of synonymous codons^21^. The ENCs of all the chloroplast genes in the six kinds of loquats range from 34 to 56, indicating diverse and relatively weak CUB in these genes. The relationship between the base composition and ENC was shown using an ENC plot. Most genes did not fall within the standard curve, indicating that the base composition did not affect the codon usage bias (Fig. 6D).

### RNA editing

Some of the transcription products (RNAs) of the chloroplast genome will be edited during posttranscriptional modification. In seed plants, RNA editing in the chloroplast often converts cytidine into uridine (C to T RNA editing)^22^. The RNA editing profile under the two distinct stresses, freezing and chilling, was examined using cultivated loquat “JFZ”. Only if the same RNA editing sites were detected in at least 2 of the 3 sample repeats would the RNA editing sites count as a true editing site. In total, 53 RNA editing sites were detected in all the samples. Most RNA editing sites were located in gene bodies (including 30 genes), and 4 RNA editing sites were located in intergenic regions. The *rpl2* contained the most RNA editing sites (6 RNA editing sites), followed by *petB*, *atpA*, *rpoA*, and *rpl23*, which contained 4, 3, 3, and 3 RNA editing sites, respectively (Table. S6). Among the 53 RNA editing sites, 29 were detected in the control group samples, 23 were in the samples under freezing stress group samples, and 50 in samples under chilling stress. The chilling samples contained 19 unique RNA editing sites, and 5 of them are located in the gene *rpl2*. We then analyzed different time points under chilling stress. We detected 6 time-specific RNA editing sites at 1 h time point, with 4 of them located in *rpl2*.

RNA editing profiles were also detected across different developmental stages (from young to old) of leaves in the “TY” wild loquat. There were a total of 35 RNA editing sites detected in “TY”. From young leaf to old leaf samples, the number of detected RNA editing sites was gradually increasing, they were 18, 24, and 27, respectively (Table. S7).

### Phylogenetic analysis

Protein coding genes from all the six kinds of loquats were used to perform Ka/Ks analysis, Ka/Ks is an index that indicates evolutionary selection. The results showed that two genes, *rpl22* and *rps12* were under positive selection. Most of the other genes were under purifying selection, and three genes, *ndhD* (*p*-value < 0.01), *atpA* (*p*-value < 0.01), and *rps14* (*p*-value < 0.05), were under significant purifying selection.

**Figure 7:**
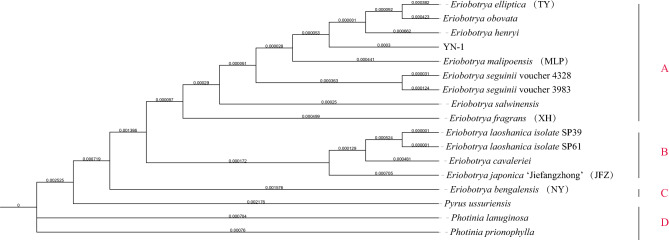
Phylogenetic tree, including 13 wild loquats, 1 cultivated loquat, *Pyrus ussuriensis*, *Photinia lanuginosa.* and *Photinia prionophylla.* The phylogenetic tree was constructed using whole chloroplast genome sequences of the 17 species of loquats (raxml-ng with parameters—model TVM+G—bs-trees 1000.).

Chloroplast genomes of 13 different kinds of wild loquats, 1 cultivated loquat, *Pyrus ussuriensis*, *Photinia lanuginosa,* and *Photinia prionophylla* were used to construct a phylogenetic tree (Table S8). Plants of *Eriobotrya* were separated from the other two genera, while among the *Eriobotrya*, wild loquats and cultivated loquat ‘JFZ’ were divided into three groups. A group contained most of the wild loquats we used, B groups included two *E. laoshanica* isolates, *E. cavaleriei*, and cultivated loquat--*Eriobotrya japonica ‘*JFZ’, C group only contained *E. bengalensis*. As shown in Fig. 7, the unknown wild loquat “YN-1” is closely related to *E. malipoensis* (MLP) and *E. henryi*, followed by *E. elliptica* (TY) and *E. obovata*.

## Materials and methods

### Sample sequencing, and chloroplast genome assembly

The leaves of the wild loquats (“YN-1” and “TY”) and cultivated loquat ‘Jiefangzhong’ (JFZ) were collected from trees planted on the Putian University campus located on Longqiao Street, Putian, China. The samples were sent to Biomark Company for DNA extraction, library construction, and second-generation sequencing (Illumina novaseq 6000 paired end sequencing, 150 bp). The chloroplast genomes of both “YN-1” and “TY” were assembled using GetOrganelle v1.7.6.1^23^ with defined parameters and annotated using CPGAVAS2^24^ (based on the ‘43-plastome’ dataset). The results were manually corrected. The final gbf files were uploaded to OGDRAW^25^ for chloroplast genome map drawing.

Collection of plant material comply with relevant institutional, national, and international guidelines and legislation.

### Chloroplast genome comparative analysis

The chloroplast genomes of wild loquats *Eriobotrya malipoensis* (MLP), *Eriobotrya bengalensis* (NY), and *Eriobotrya fragrans* (XH) were downloaded from the Lauraceae Chloroplast Genome Database (https://lcgdb.wordpress.com/). All the chloroplast genomes’ sequences, including the three newly assembled ones, were aligned using the MAFFT^26^ software (Unweighted Pair Group Method with Arithmetic Mean), The result was then uploaded to DnaSP6.0^27^ for DNA polymorphism analysis. GC content and codon usage bias analysis were performed using EMBOSS-6.6.0^28^ and CodonW software(https://codonw.sourceforge.net/, defined parameter). Codon adaptation index (CAI) was performed on the GALAXY platform^29^. The mVISTA^30^ was used to do a comparative genomics analysis. MISA^31^(misa.ini parmeter: 1–10, 2–5, 3–4, 4–3, 5–3, 6–3) and REPuter^32^ (minimal repeat size, 18 bp) were used to analyze SSR and repeats. Single nucleotide polymorphisms and indels were detected using DnaSP6.0 and a Python script (https://www.biostars.org/p/119214/). Non-synonymous and synonymous rates of substitution analysis were conducted using ParaAT 2.0^33^ and KaKs_Calculator 2.0^34^. All related figures were drawn using R and OFFICE.

### SNP calling for RNA editing site

After being treated to −3 °C (freezing) and −4 °C (chilling), leaf samples from “JFZ” were gathered and sent for RNA-seq in Metware Biotechnology Inc. (Illumina novaseq 6000 paired-end sequencing, 150 bp). SNP calling for RNA editing site detection was performed using RNA-seq data of wild loquat “TY” and cultivated loquat ‘JFZ’. The paired-end reads were mapped to the chloroplast genome using HISAT2 and sorted using samtools^35,36^. SNP calling was performed using samtools (mpileup and bcftools) and filleted with parameters: quality >= 20 and depth > 10. SNPs in at least two replications were selected as the final RNA editing candidate sites.

### Phylogenetic analysis

All the chloroplast genomes of wild loquats, except the 3 members used in the comparison step were downloaded from NCBI database (https://www.ncbi.nlm.nih.gov/), along with chloroplast genomes of *Pyrus ussuriensis*, *Photinia lanuginosa,* and *Photinia prionophylla*. All the chloroplast sequences were aligned using MAFFT, and a phylogenetic tree was constructed using raxml-ng^37^ with parameters --model TVM+G --bs-trees 1000.

## Discussion

Two novel chloroplast genomes, TY and YN-1, were assembled in this research. Their sizes are 159,471 bp and 159,399 bp, respectively. The findings of the gene contents analysis showed that there are 83 protein-coding genes, 8 rRNA, and 36 tRNA in these two wild loquats. This is not the same as the XH loquat, which is devoid of the *psbK* gene. Although the *psbK* gene is one of the genes related to PS II, reports suggest that *psbK* is not crucial for PS II activity in *Synechocytis*^38^.

Simple sequence repeats (SSRs), typically consisting of 1 to 6 bp repeat units, are important genetic markers that are widely used in evolutionary and population genetics studies^39,40^. Here, we conducted an SSR analysis on 6 kinds of loquats, the “XH” loquat contained the highest number of SSRs. Two types of SSRs, “AGC/CTG” and “AAAG/CTTT”, were exclusively identified in “XH”, while “AAT/ATT” was only detected in “TY”. These species-specific SSRs can be used in species differentiation analysis^41^. In the repetitive sequences analysis, most of the repetitive sequences range from 25 to 50 bp. The “MLP” loquat is an exception; most repetitive sequences contained were more than 70 bp, indicating that rearrangement events may be more intense in “MLP”. It has been reported that repetitive sequences are associated with rearrangement endpoints^42^.

RNA editing in chloroplast genes has been reported in several studies^43,44^. There is a report also showing that RNA editing profiles changed under stress situation^10^. Here, we did detect several RNA editing sites in some genes, using RNA_seq data of cultivating loquat ‘Jiefangzhong’, like *rpl2*, *petB*, *atpA*, *rpoA*, and *rpl23* et al. *rpl2* gene seems to play an important role when ‘Jiefangzhong’ response to chilling stress, there were 6 RNA editing sites detected in *rpl2*. *rpl2* is related to plasmid development, and mutant-caused editing defective in *rpl2* showed abnormal phenotype during cold stress^45,46^. RNA editing was also performed in “TY” loquat, results showed that the number of RNA editing sites was gradually increased in leaves from young to mature, and stage-specific RNA editing sites were also found, indicating that RNA editing dynamic in different development stages of leaves. In *Coprinopsis cinerea*, stage-specific patterns and dynamic RNA editing sites were also detected^47^.

In the phylogenetic analysis, “TY” (*E. elliptica*) was close to *E. obovata*, and then *E. henryi*. The “YN-1” is also very close to “TY”, indicating a close relationship among these species of wild loquats. Based on these findings, we can infer that “YN-1” is a new wild loquat. “NY” (*E. bengalensis*) loquat is the only wild loquat that was separated from other wild loquats, suggesting an origin from a different region. There is a report showing that sisterhood exists in *E. bengalensis* with nine other species of *Eriobotrya* in their analysis. In their phylogenetic tree, *E. bengalensis* was also separated from the other species of *Eriobotrya* they used, located in a solo branch^18^.

### Supplementary Information

Supplementary Information.

Supplementary Tables.

## Data Availability

The three new assembled chloroplast genomes can be download from link https://db.cngb.org/cnsa/ with accession code CNP0005553
