# Supplementary material for: Chloroplast genomes of Eriobotrya elliptica and an unknown wild loquat “YN-1”
Source: Sci Rep. 2024 Aug 13;14:18816. doi: 10.1038/s41598-024-69882-7 (PMC11322449; doi:10.1038/s41598-024-69882-7)
Supplement: Supplementary file 1 — Supplementary Information. [file 41598_2024_69882_MOESM1_ESM.pdf]

Chloroplast genomes of *Eriobotrya elliptica* and an unknown wild loquat "YN-1"

Zhicong Lin[1], Qing Guo[2], Shiwei Ma[1], Hailan Lin[1], Shunquan Lin[1], Shoukai Lin[1]*, Jincheng Wu[2]*

1 College of Environmental and Biological Engineering, Putian University, Fujian Provincial Key Laboratory of Ecology-Toxicological Effects & Control for Emerging Contaminants, Key Laboratory of Ecological Environment and Information Atlas (Putian University) Fujian Provincial University, Putian, 351100, China

2 College of Environmental and Biological Engineering, Putian University, 351100, China

Email: woshi0655@126.com (Z.L.); 2372010382@qq.com (Q.G); mshiwei@163.com (S.M.); linhailan.ok@163.com (H.L), loquat@scau.edu.cn (Shunquan. L.).

*Correspondence: linshoukai@ptu.edu.cn (Shoukai L.); wjc2384@163.com (J.W.).

Supplemental Table legend (Please see Detail in Supplemental Table)

Table S1. Nucleotide diversity among six different kinds of loquat (window length: 600 bp, step size: 200 bp).

Table S2. SNPs and Indels among six kinds of loquat (Including YN-1, XH, ‘JFZ’, MLP, NY and TY)

Table S3. SSR in different kinds of loquats (Including YN-1, XH, ‘JFZ’, MLP, NY and TY)

Table S4. Repetitive sequence in different kinds of loquats (Including YN-1, XH, ‘JFZ’, MLP, NY and TY)

Table S5. Relative Synonymous Codon Usage Calculation (RSCU) values in different kinds of loquat (Including YN-1, XH, ‘JFZ’, MLP, NY and TY)

Table S6. RNA editing sites’ information of loquat ‘JFZ’. CK means control, CT-2, CT-4, CT-6 represent loquat treated in -3℃ for 2 h, 4 h and 6 h. LT means loquat being treated in 4℃ for different times (from 0.5 h to 24 h).

Table S7. RNA editing sites’ information of TY loquat. TY-1 to TY-3 means loquats from young to mature.

Table S8. ID of different species used in phylogenetic analysis

Supplemental Table

Figure.S1

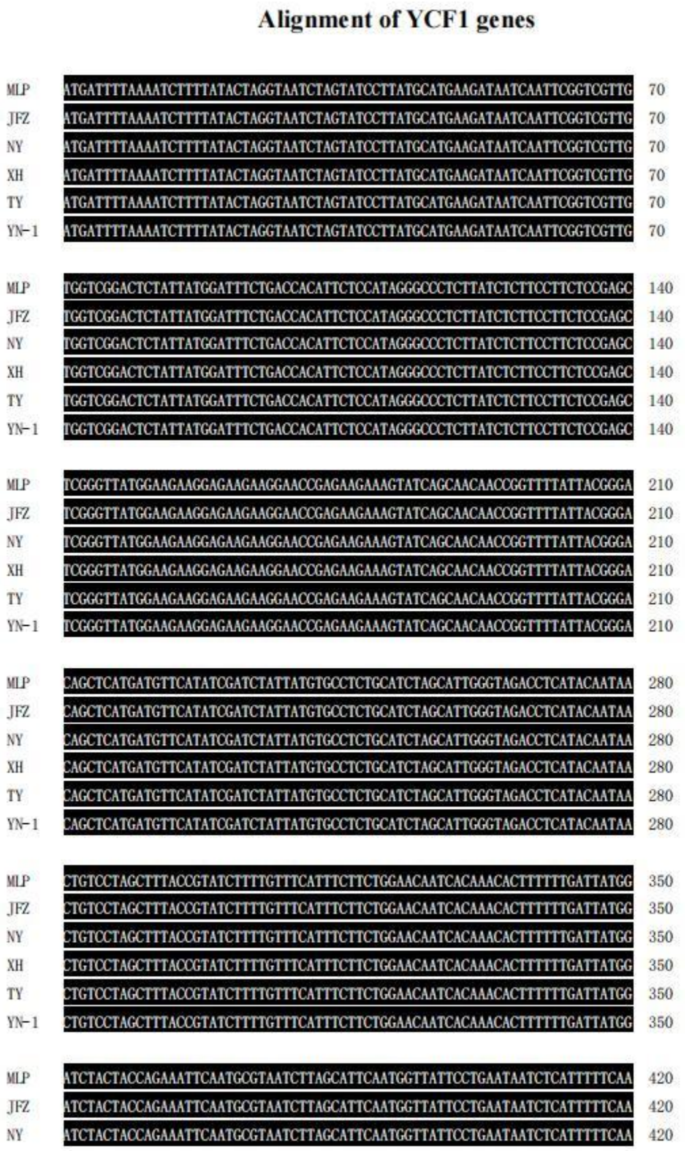

```
XH     ATCTACTACCAGAAATTCAATGCGTAATCTTAGCATTCAATGGTTATTCCTGAATAATCTCATTTTTCAA  420
TY     ATCTACTACCAGAAATTCAATGCGTAATCTTAGCATTCAATGGTTATTCCTGAATAATCTCATTTTTCAA  420
YN-1   ATCTACTACCAGAAATTCAATGCGTAATCTTAGCATTCAATGGTTATTCCTGAATAATCTCATTTTTCAA  420

MLP    TTATTCAACCATTTCATTTTACCAAGTTCAATGTTAGTCAGATTAGTCAACATTTATATGTTTCGATGCA  490
JFZ    TTATTCAACCATTTCATTTTACCAAGTTCAATGTTAGTCAGATTAGTCAACATTTATATGTTTCGATGCA  490
NY     TTATTCAACCATTTCATTTTACCAAGTTCAATGTTAGTCAGATTAGTCAACATTTATATGTTTCGATGCA  490
XH     TTATTCAACCATTTCATTTTACCAAGTTCAATGTTAGTCAGATTAGTCAACATTTATATGTTTCGATGCA  490
TY     TTATTCAACCATTTCATTTTACCAAGTTCAATGTTAGTCAGATTAGTCAACATTTATATGTTTCGATGCA  490
YN-1   TTATTCAACCATTTCATTTTACCAAGTTCAATGTTAGTCAGATTAGTCAACATTTATATGTTTCGATGCA  490

MLP    ACAACAAGATGTTATTTGTAACAAGTAGTTTTGTTGGTTGGTTAATTGGTCACATTTTATTCATGAAATG  560
JFZ    ACAACAAGATGTTATTTGTAACAAGTAGTTTTGTTGGTTGGTTAATTGGTCACATTTTATTCATGAAATG  560
NY     ACAACAAGATGTTATTTGTAACAAGTAGTTTTGTTGGTTGGTTAATTGGTCACATTTTATTCATGAAATG  560
XH     ACAACAAGATGTTATTTGTAACAAGTAGTTTTGTTGGTTGGTTAATTGGTCACATTTTATTCATGAAATG  560
TY     ACAACAAGATGTTATTTGTAACAAGTAGTTTTGTTGGTTGGTTAATTGGTCACATTTTATTCATGAAATG  560
YN-1   ACAACAAGATGTTATTTGTAACAAGTAGTTTTGTTGGTTGGTTAATTGGTCACATTTTATTCATGAAATG  560

MLP    GGTTGGGTTGGTATTAGGCTGGATACAGCAAAATAATTCTATTAGATCTAATGTACTTATTAGATCTAAT  630
JFZ    GGTTGGGTTGGTATTAGGCTGGATACAGCAAAATAATTCTATTAGATCTAATGTACTTATTAGATCTAAT  630
NY     GGTTGGGTTGGTATTAGGCTGGATACAGCAAAATAATTCTATTAGATCTAATGTACTTATTAGATCTAAT  630
XH     GGTTGGGTTGGTATTAGGCTGGATACAGCAAAATAATTCTATTAGATCTAATGTACTTATTAGATCTAAT  630
TY     GGTTGGGTTGGTATTAGGCTGGATACAGCAAAATAATTCTATTAGATCTAATGTACTTATTAGATCTAAT  630
YN-1   GGTTGGGTTGGTATTAGGCTGGATACAGCAAAATAATTCTATTAGATCTAATGTACTTATTAGATCTAAT  630

MLP    AAGTACCTTGTGTCAGAATTGAGAAATTCTATGGCTCGAATCTTTAGTATTCTCTTATTTATTACCTGTG  700
JFZ    AAGTACCTTGTGTCAGAATTGAGAAATTCTATGGCTCGAATCTTTAGTATTCTCTTATTTATTACCTGTG  700
NY     AAGTACCTTGTGTCAGAATTGAGAAATTCTATGGCTCGAATCTTTAGTATTCTCTTATTTATTACCTGTG  700
XH     AAGTACCTTGTGTCAGAATTGAGAAATTCTATGGCTCGAATCTTTAGTATTCTCTTATTTATTACCTGTG  700
TY     AAGTACCTTGTGTCAGAATTGAGAAATTCTATGGCTCGAATCTTTAGTATTCTCTTATTTATTACCTGTG  700
YN-1   AAGTACCTTGTGTCAGAATTGAGAAATTCTATGGCTCGAATCTTTAGTATTCTCTTATTTATTACCTGTG  700

MLP    TCTACTATTTAGGCAGAATACCGTCACCCATTGTTACTAAGAAACTGAAAGAAACCTCAGAAACGGAAGA  770
JFZ    TCTACTATTTAGGCAGAATACCGTCACCCATTGTTACTAAGAAACTGAAAGAAACCTCAGAAACGGAAGA  770
NY     TCTACTATTTAGGCAGAATACCGTCACCCATTGTTACTAAGAAACTGAAAGAAACCTCAGAAACGGAAGA  770
XH     TCTACTATTTAGGCAGAATACCGTCACCCATTGTTACTAAGAAACTGAAAGAAACCTCAGAAACGGAAGA  770
TY     TCTACTATTTAGGCAGAATACCGTCACCCATTGTTACTAAGAAACTGAAAGAAACCTCAGAAACGGAAGA  770
YN-1   TCTACTATTTAGGCAGAATACCGTCACCCATTGTTACTAAGAAACTGAAAGAAACCTCAGAAACGGAAGA  770

MLP    AAGGGGGGAAAGTGAGGAAGAAACAGATGTAGAAATAGAAACAACTTCCGAAACGAAGGGGACTAAACAG  840
JFZ    AAGGGGGGAAAGTGAGGAAGAAACAGATGTAGAAATAGAAACAACTTCCGAAACGAAGGGGACTAAACAG  840
```

NY	AAGGGGGGAAAGTGAGGAAGAAACAGATGTAGAAATAGAAACAACTTCCGAAACGAAGGGGACTAAACAG	840
XH	AAGGGGGGAAAGTGAGGAAGAAACAGATGTAGAAATAGAAACAACTTCCGAAACGAAGGGGACTAAACAG	840
TY	AAGGGGGGAAAGTGAGGAAGAAACAGATGTAGAAATAGAAACAACTTCCGAAACGAAGGGGACTAAACAG	840
YN-1	AAGGGGGGAAAGTGAGGAAGAAACAGATGTAGAAATAGAAACAACTTCCGAAACGAAGGGGACTAAACAG	840
MLP	GAACAAGAGGGATCCACCGAAGAAGATCCTTCCCCTTCCCTTTTTTCGGAAGAAAAGGAGGATACGGACA	910
JFZ	GAACAAGAGGGATCCACCGAAGAAGATCCTTCCCCTTCCCTTTTTTCGGAAGAAAAGGAGGATACGGACA	910
NY	GAACAAGAGGGATCCACCGAAGAAGATCCTTCCCCTTCCCTTTTTTCGGAAGAAAAGGAGGATACGGACA	910
XH	GAACAAGAGGGATCCACCGAAGAAGATCCTTCCCCTTCCCTTTTTTCGGAAGAAAAGGAGGATACGGACA	910
TY	GAACAAGAGGGATCCACCGAAGAAGATCCTTCCCCTTCCCTTTTTTCGGAAGAAAAGGAGGATACGGACA	910
YN-1	GAACAAGAGGGATCCACCGAAGAAGATCCTTCCCCTTCCCTTTTTTCGGAAGAAAAGGAGGATACGGACA	910
MLP	AAATCGATGAAACGAAAGAGATCCGAGTGAATGGAAAGGAAAAAACAAAGGATCAATTCCACTTTAAAGA	980
JFZ	AAATCGATGAAACGAAAGAGATCCGAGTGAATGGAAAGGAAAAAACAAAGGATCAATTCCACTTTAAAGA	980
NY	AAATCGATGAAACGAAAGAGATCCGAGTGAATGGAAAGGAAAAAACAAAGGATCAATTCCACTTTAAAGA	980
XH	AAATCGATGAAACGAAAGAGATCCGAGTGAATGGAAAGGAAAAAACAAAGGATCAATTCCACTTTAAAGA	980
TY	AAATCGATGAAACGAAAGAGATCCGAGTGAATGGAAAGGAAAAAACAAAGGATCAATTCCACTTTAAAGA	980
YN-1	AAATCGATGAAACGAAAGAGATCCGAGTGAATGGAAAGGAAAAAACAAAGGATCAATTCCACTTTAAAGA	980
MLP	AACACGGTATAAAAAAAGACCCGTTTATGAAACTTATTATCTGGATGGGAATCAAGAACAAGAAAATTCA	1050
JFZ	AACACGGTATAAAAAAAGACCCGTTTATGAAACTTATTATCTGGATGGGAATCAAGAACAAGAAAATTCA	1050
NY	AACACGGTATAAAAAAAGACCCGTTTATGAAACTTATTATCTGGATGGGAATCAAGAACAAGAAAATTCA	1050
XH	AACACGGTATAAAAAAAGACCCGTTTATGAAACTTATTATCTGGATGGGAATCAAGAACAAGAAAATTCA	1050
TY	AACACGGTATAAAAAAAGACCCGTTTATGAAACTTATTATCTGGATGGGAATCAAGAACAAGAAAATTCA	1050
YN-1	AACACGGTATAAAAAAAGACCCGTTTATGAAACTTATTATCTGGATGGGAATCAAGAACAAGAAAATTCA	1050
MLP	AAGTTAGAAATATTAAAAAAAAGAGATATCTTCTGGTTTGAAAAACCAATTGTGACTATTCTTTTCGACT	1120
JFZ	AAGTTAGAAATATTAAAAAAAAGAGATATCTTCTGGTTTGAAAAACCAATTGTGACTATTCTTTTCGACT	1120
NY	AAGTTAGAAATATTAAAAAAAAGAGATATCTTCTGGTTTGAAAAACCTATTGTGACTATTCTTTTCGACT	1120
XH	AAGTTAGAAATATTAAAAAAAAGAGATATCTTCTGGTTTGAAAAACCAATTGTGACTATTCTTTTCGACT	1120
TY	AAGTTAGAAATATTAAAAAAAAGAGATATCTTCTGGTTTGAAAAACCAATTGTGACTATTCTTTTCGACT	1120
YN-1	AAGTTAGAAATATTAAAAAAAAGAGATATCTTCTGGTTTGAAAAACCAATTGTGACTATTCTTTTCGACT	1120
MLP	ATAAACGTTGGAACCGTCCGTTACGATATATAAAAAACAGTCGATTTGAAAAGTCTGTAAGAAACGAAAT	1190
JFZ	ATAAACGTTGGAACCGTCCGTTACGATATATAAAAAACAGTCGATTTGAAAAGTCTGTAAGAAACGAAAT	1190
NY	ATAAACGTTGGAACCGTCCGTTACGATATATAAAAAACAGTCGATTTGAAAAGTCTGTAAGAAATGAAAT	1190
XH	ATAAACGTTGGAACCGTCCGTTACGATATATAAAAAACAGTCGATTTGAAAAGTCTGTAAGAAACGAAAT	1190
TY	ATAAACGTTGGAACCGTCCGTTACGATATATAAAAAACAGTCGATTTGAAAAGTCTGTAAGAAACGAAAT	1190
YN-1	ATAAACGTTGGAACCGTCCGTTACGATATATAAAAAACAGTCGATTTGAAAAGTCTGTAAGAAACGAAAT	1190
MLP	GTCACAATATTTTTTTTCTACATGTCAAAGTGATGAAAAAGAAAGAATATCTTTTACGTACCCACCCAGT	1260

```
JFZ    GTCACAATATTTTTTTTCTACATGTCAAAGTGATGAAAAAGAAAGAATATCTTTTACGTACCCACCCAGT    1260
NY     GTCACAATATTTTTTTTCTACATGTCAAAGTGATGAAAAAGAAAGAATATCTTTTACGTACCCACCCAGT    1260
XH     GTCACAATATTTTTTTTCTACATGTCAAAGTGATGAAAAAGAAAGAATATCTTTTACGTACCCACCCAGT    1260
TY     GTCACAATATTTTTTTTCTACATGTCAAAGTGATGAAAAAGAAAGAATATCTTTTACGTACCCACCCAGT    1260
YN-1   GTCACAATATTTTTTTTCTACATGTCAAAGTGATGAAAAAGAAAGAATATCTTTTACGTACCCACCCAGT    1260

MLP    TTAGCAACTTTTTTTGAAATGATACAAAGAAAGATGTCTCTGTTCACAAAGAAACATTCCCCTCTAATG    1330
JFZ    TTAGCAACTTTTTTTGAAATGATACAAAGAAAGATGTCTCTGTTCACAAAGAAACATTCCCCTCTAATG    1330
NY     TTAGCAACTTTTTTTGAAATAATACAAAGAAAGATGTCTCTGTTCACAAAGAAACATTCCCCTCTAATG    1330
XH     TTAGCAACTTTTTTTGAAATGATACAAAGAAAGATGTCTCTGTTCACAAAGAAACATTCCCCTCTAATG    1330
TY     TTAGCAACTTTTTTTGAAATGATACAAAGAAAGATGTCTCTGTTCACAAAGAAACATTCCCCTCTAATG    1330
YN-1   TTAGCAACTTTTTTTGAAATGATACAAAGAAAGATGTCTCTGTTCACAAAGAAACATTCCCCTCTAATG    1330

MLP    AATTTTATAATCATTGGAGTTATACCAACGAACATAAAAAGAAAAACCTAAGCAAAAAAATTTTTAATAG    1400
JFZ    AATTTTATAATCATTGGAGTTATACCAACGAACATAAAAAGAAAAACCTAAGCAAAAAAATTTTTAATAG    1400
NY     AATTTTATAATCATTGGAGTTATACCAACGAACATAAAAAGAAAAACCTAAGCAAAAAAATTTTTAATAG    1400
XH     AATTTTATAATCATTGGAGTTATACCAACGAACATAAAAAGAAAAACCTAAGCAAAAAAATTTTTAATAG    1400
TY     AATTTTATAATCATTGGAGTTATACCAACGAACATAAAAAGAAAAACCTAAGCAAAAAAATTTTTAATAG    1400
YN-1   AATTTTATAATCATTGGAGTTATACCAACGAACATAAAAAGAAAAACCTAAGCAAAAAAATTTTTAATAG    1400

MLP    AGTAAAAACTATCGACAAAATTCTAGATAAGGAAGCCCTTGTTATGAATGTACTCGAAAAAAGAACTAGA    1470
JFZ    AGTAAAAACTATCGACAAAATTCTAGATAAGGAAGCCCTTGTTATGAATGTACTCGAAAAAAGAACTAGA    1470
NY     AGTAAAAACTATCGACAAAATTCTAGATAAGGAAGCCCTTGTTATGAATGTACTCGAAAAAAGAACTAGA    1470
XH     AGTAAAAACTATCGACAAAATTCTAGATAAGGAAGCCCTTGTTATGAATGTACTCGAAAAAAGAACTAGA    1470
TY     AGTAAAAACTATCGACAAAATTCTAGATAAGGAAGCCCTTGTTATGAATGTACTCGAAAAAAGAACTAGA    1470
YN-1   AGTAAAAACTATCGACAAAATTCTAGATAAGGAAGCCCTTGTTATGAATGTACTCGAAAAAAGAACTAGA    1470

MLP    TTGTGTACTGATAAGACTAAAAAAGAATACTTACCAAAAATATATGATCCTTTCGTCAATGGACCCTACC    1540
JFZ    TTGTGTACTGATAAGACTAAAAAAGAATACTTACCAAAAATATATGATCCTTTCGTCAATGGACCCTACC    1540
NY     TTGTGTACTGATAAGACTAAAAACGAATACTTACCAAAAATATATGATCCTTTCGTCAATGGACCCTACC    1540
XH     TTGTGTACTGATAAGACTAAAAAAGAATACTTACCAAAAATATATGATCCTTTCGTCAATGGACCCTACC    1540
TY     TTGTGTACTGATAAGACTAAAAAAGAATACTTACCAAAAATATATGATCCTTTCGTCAATGGACCCTACC    1540
YN-1   TTGTGTACTGATAAGACTAAAAAAGAATACTTACCAAAAATATATGATCCTTTCGTCAATGGACCCTACC    1540

MLP    GCGGACGAATCAAAAAATTGTTTACACTTTCAAGCATAAATGAAGCTTCTGTAAAAAATTATAAGATTTG    1610
JFZ    GCGGACGAATCAAAAAATTGTTTACACTTTCAAGCATAAATGAAGCTTCTGTAAAAAATTATAAGATTTG    1610
NY     GCGGACGAATCAAAAAATTGTTTACACTTTCAAGCATAAATGAAGCTTCTGTAAAAAATTACAAGATTTG    1610
XH     GCGGACGAATCAAAAAATTGTTTACACTTTCAAGCATAAATGAAGCTTCTGTAAAAAATTCTAAGATTTG    1610
TY     GCGGACGAATCAAAAAATTGTTTACACTTTCAAGCATAAATGAAGCTTCTGTAAAAAATTATAAGATTTG    1610
YN-1   GCGGACGAATCAAAAAATTGTTTACACTTTCAAGCATAAATGAAGCTTCTGTAAAAAATTATAAGATTTG    1610
```

```
MLP    GATAAATAAAATTCATGGTATCCTTCTTATTAGTAATTACTTAGAATTTGAACAAAAATAAATTCATTT  1680
JFZ    GATAAATAAAATTCATGGTATCCTTCTTATTAGTAATTACTTAGAATTTGAACAAAAAGAAATTCATTT  1680
NY     GATAAATAAAATTCATGGTATCCTTCTTATTATTAATTACTTAGAATTTGAACAAAAATAAATTCATTT  1680
XH     GATAAATAAAATTCATGGTATCCTTCTTATTAGTAATTACTTAGAATTTGAACAAAAATAAATTCATTT  1680
TY     GATAAATAAAATTCATGGTATCCTTCTTATTAGTAATTACTTAGAATTTGAACAAAAATAAATTCATTT  1680
YN-1   GATAAATAAAATTCATGGTATCCTTCTTATTAGTAATTACTTAGAATTTGAACAAAAATAAATTCATTT  1680

MLP    GATAGAAAATCATTAACAACAGAAATTTTTTATTTATTAAATTTAATCAATGAATTGGTTGTAAAATACA  1750
JFZ    GATAGAAAATCATTAACAACAGAAATTTTTTATTTATTAAATTTAATCAATGAATTGGTTGTAAAATACA  1750
NY     GATAGAAAATCATTAACAACAGAAATTTTTTATTTATTAAATTTAATCAATGAATTGGTTGTAAAATACA  1750
XH     GATAGAAAATCATTAACAACAGAAATTTTTTATTTATTAAATTTAATCAATGAATTGGTTGTAAAATACA  1750
TY     GATAGAAAATCATTAACAACAGAAATTTTTTATTTATTAAATTTAATCAATGAATTGGTTGTAAAATACA  1750
YN-1   GATAGAAAATCATTAACAACAGAAATTTTTTATTTATTAAATTTAATCAATGAATTGGTTGTAAAATACA  1750

MLP    CATCAAATTTAAATTTTAAAAGACTTTTTTTAGTTACAGAACACGAACAAGTAAGAATTGATTCAGAGGA  1820
JFZ    CATCAAATTTAAATTTTAAAAGACTTTTTTTAGTTACAGAACACGAACAAGTAAGAATTGATTCAGAGGA  1820
NY     CATCAAATTTCAATTTTAAAAGACTTTTTTTAGTTACAGAACACGAACAAGTAAGAATTGATTCAGAGGA  1820
XH     CATCAAATTTAAATTTTAAAAGACTTTTTTTAGTTACAGAACACGAACAAGTAAGAATTGATTCAGAGGA  1820
TY     CATCAAATTTAAATTTTAAAAGACTTTTTTTAGTTACAGAACACGAACAAGTAAGAATTGATTCAGAGGA  1820
YN-1   CATCAAATTTAAATTTTAAAAGACTTTTTTTAGTTACAGAACACGAACAAGTAAGAATTGATTCAGAGGA  1820

MLP    TCGAATCCAAATTTTAAAATTATTATTTGATGCAATTAGAACTGTTCCCAACGATAAAATGATTAAAAAA  1890
JFZ    TCGAATCCAAATTTTAAAATTATTATTTGATGCAATTAGAACTGTTCCCAACGATAAAATGATTAAAAAA  1890
NY     TCGAATCCAAATTTTAAAATTATTATTTGATGCAATTAGAACTGTTCCCAACGATAAAATGATTAAAAAA  1890
XH     TCGAATCCAAATTTTAAAATTATTATTTGATGCAATTAGAACTGTTCCCAACGATAAAATGATTAAAAAA  1890
TY     TCGAATCCAAATTTTAAAATTATTATTTGATGCAATTAGAACTGTTCCCAACGATAAAATGATTAAAAAA  1890
YN-1   TCGAATCCAAATTTTAAAATTATTATTTGATGCAATTAGAACTGTTCCCAACGATAAAATGATTAAAAAA  1890

MLP    AAATCTATTGGAATAAAAGAAATTAATAAAAAAGTTCCTCGATGGTCATACAAATTAATCAACGATTTTG  1960
JFZ    AAATCTATTGGAATAAAAGAAATTAATAAAAAAGTTCCTCGATGGTCATACAAATTAATCAACGATTTTG  1960
NY     AAATCTATTGGAATAAAAGAAATTAATAAAAAAGTTCCTCGATGGTCATACAAATTAATCAACGATTTTG  1960
XH     AAATCTATTGGAATAAAAGAAATTAATAAAAAAGTTCCTCGATGGTCATACAAATTAATCAACGATTTTG  1960
TY     AAATCTATTGGAATAAAAGAAATTAATAAAAAAGTTCCTCGATGGTCATACAAATTAATCAACGATTTTG  1960
YN-1   AAATCTATTGGAATAAAAGAAATTAATAAAAAAGTTCCTCGATGGTCATACAAATTAATCAACGATTTTG  1960

MLP    AACAACAGGAGGGGGAAACCGAAGAAACTGTGGTAGAGGATCATCAGATTCGTTCAAGAAAATCCAAACG  2030
JFZ    AACAACAGGAGGGGGAAACCGAAGAAACTGTGGTAGAGGATCATCAGATTCGTTCAAGAAAATCCAAACG  2030
NY     AACAACAGGAGGGGGAAACCGAAGAAACTGTGGTAGAGGATCATCAGATTCGTTCAAGAAAATCCAAACG  2030
XH     AACAACAGGAGGGGGAAACCGAAGAAACTGTGGTAGAGGATCATCAGATTCGTTCAAGAAAATCCAAACG  2030
TY     AACAACAGGAGGGGGAAACCGAAGAAACTGTGGTAGAGGATCATCAGATTCGTTCAAGAAAATCCAAACG  2030
YN-1   AACAACAGGAGGGGGAAACCGAAGAAACTGTGGTAGAGGATCATCAGATTCGTTCAAGAAAATCCAAACG  2030
```

MLP	TGTAGTGATTTTTACTGATAACCAACAAAATACTGATGCTTATACTAATACCAAAGAAACTAATAATACT	2100
JFZ	TGTAGTGATTTTTACTGATAACCAACAAAATACTGATGCTTATACTAATACCAAAGAAACTAATAATACT	2100
NY	TGTAGTGATTTTTACTGATAACCAACAAAATACTGATGCTTATACTAATACCAAAGAAACTAATAATACT	2100
XH	TGTAGTGATTTTTACTGATAACCAACAAAATACTGATGCTTATACTAATACCAAAGAAACAAATAATACT	2100
TY	TGTAGTGATTTTTACTGATAACCAACAAAATACTGATGCTTATACTAATACCAAAGAAACTAATAATACT	2100
YN-1	TGTAGTGATTTTTACTGATAACCAACAAAATACTGATGCTTATACTAATACCAAAGAAACTAATAATACT	2100
MLP	GATCAAACAGGCGAAGTTGCTTTGATACGTTATTCCCAACAATCGGATTTTCGTCGAGACATAATCAAAG	2170
JFZ	GATCAAACAGGCGAAGTTGCTTTGATACGTTATTCCCAACAATCGGATTTTCGTCGAGACATAATCAAAG	2170
NY	GATCAAACAGGCGAAGTTGCTTTGATACGTTATTCCCAACAATCGGATTTTCGTCGAGACATAATCAAAG	2170
XH	GATCAAACAGGCGAAGTTGCTTTGATACGTTATTCCCAACAATCGGATTTTCGTCGAGACATAATCAAAG	2170
TY	GATCAAACAGGCGAAGTTGCTTTGATACGTTATTCCCAACAATCGGATTTTCGTCGAGACATAATCAAAG	2170
YN-1	GATCAAACAGGCGAAGTTGCTTTGATACGTTATTCCCAACAATCGGATTTTCGTCGAGACATAATCAAAG	2170
MLP	GCTCCATGCGCGCTCAAAGACGTAAAACAGTTACTTGGAAACTCTTTGAAGCAAATGCGCATTCCCCTCT	2240
JFZ	GCTCCATGCGCGCTCAAAGACGTAAAACAGTTACTTGGAAACTCTTTGAAGCAAATGCGCATTCCCCTCT	2240
NY	GCTCCATGCGCGCTCAAAGACGTAAAACAGTTACTTGGAAACTCTTTGAAGCAAATGCGCATTCCCCTCT	2240
XH	GCTCCATGCGCGCTCAAAGACGTAAAACAGTTACTTGGAAACTCTTTGAAGCAAATGCGCATTCCCCTCT	2240
TY	GCTCCATGCGCGCTCAAAGACGTAAAACAGTTACTTGGAAACTCTTTGAAGCAAATGCGCATTCCCCTCT	2240
YN-1	GCTCCATGCGCGCTCAAAGACGTAAAACAGTTACTTGGAAACTCTTTGAAGCAAATGCGCATTCCCCTCT	2240
MLP	TTTTTTGGACCGAATAGACAAATCTTTTTTTTTTTTTTTTGATATTTCTGAACGGATGAAAAAAATTTTT	2310
JFZ	TTTTTTGGACCGAATAGACAAATCTTTTTTTTTTTTTTTTGATATTTCTGAACGGATGAAAAAAATTTTT	2310
NY	TTTTTTGGACCGAATAGACAAATCTTTTTTTTTTTTTTTTGATATTTCTGAACGGATGAAAAAAATTTTT	2310
XH	TTTTTTGGACCGAATAGACAAATCTTTTTTTTTTTTTTTTGATATTTCTGAACGGATGAAAAAAATTTTA	2310
TY	TTTTTTGGACCGAATAGACAAATCTTTTTTTTTTTTTTTTGATATTTCTGAACGGATGAAAAAAATTTTT	2310
YN-1	TTTTTTGGACCGAATAGACAAATCTTTTTTTTTTTTTTTTGATATTTCTGAACGGATGAAAAAAATTTTT	2310
MLP	AAAATTGGATGTGGAAAAACACAGAATTCACAATTTCGAATTATACAGAGAAAAAGACAAAAGAAAGCG	2380
JFZ	AAAATTGGATGTGGAAAAACACAGAATTCACAATTTCGAATTATACAGAGAAAAAGACAAAAGAAAGCG	2380
NY	AGAATTGGATGTGGAAAAACACAGAATTCACAATTTCGAATTATACAGAGAAAAAGACAAAAGAAAGCG	2380
XH	AAAATTGGATGTGGAAAAACACAGAATTCACAATTTCGAATTATACAGAGAAAAAGACAAAAGAAAGCG	2380
TY	AAAATTGGATGTGGAAAAACACAGAATTCACAATTTCGAATTATACAGAGAAAAAGACAAAAGAAAGCG	2380
YN-1	AAAATTGGATGTGGAAAAACACAGAATTCACAATTTCGAATTATACAGAGAAAAAGACAAAAGAAAGCG	2380
MLP	CGAAAAAAAAAGAGGAGGACAAAAAAGAAGAAGACAAAAGAAAGGAGAAAGTGCGTATACAAATAGCGGA	2450
JFZ	CGAAAAAAAAAGAGGAGGACAAAAAAGAAGAAGACAAAAGAAAGGAGAAAGTGCGTATACAAATAGCGGA	2450
NY	CGAAAAAAAAAGAGGAGGACAAAAAAGAAGAAGACAAAAGAAAGGAGAAAGTGCGTATACAAATAGCGGA	2450
XH	CGAAAAAAAAAGAGGAGGACAAAAAAGAAGAAGACAAAAGAAAGGAGAAAGTGCGTATACAAATAGCGGA	2450
TY	CGAAAAAAAAAGAGGAGGACAAAAAAGAAGAAGACAAAAGAAAGGAGAAAGTGCGTATACAAATAGCGGA	2450

```
YN-1   CGAAAAAAAAAGAGGAGGACAAAAAAGAAGAAGACAAAAGAAAGGAGAAAGTGCGTATACAAATAGCGGA   2450

MLP    AGCCTGGGATAGTATTTTACTTGCTCAAGTAATGAGAGGTTTTTTATTAGTAACCCAATCAATTCTTAGA   2520
JFZ    AGCCTGGGATAGTATTTTACTTGCTCAAGTAATGAGAGGTTTTTTATTAGTAACCCAATCAATTCTTAGA   2520
NY     AGCCTGGGATAGTCTTTTACTTGCTCAAGTAATGAGAGGTTTTTTATTAGTAACCCAATCAATTCTTAGA   2520
XH     AGCCTGGGATAGTATTTTACTTGCTCAAGTAATGAGAGGTTTTTTATTAGTAACCCAATCAATTCTTAGA   2520
TY     AGCCTGGGATAGTATTTTACTTGCTCAAGTAATGAGAGGTTTTTTATTAGTAACCCAATCAATTCTTAGA   2520
YN-1   AGCCTGGGATAGTATTTTACTTGCTCAAGTAATGAGAGGTTTTTTATTAGTAACCCAATCAATTCTTAGA   2520

MLP    AAATATATTATATTACCTTCATTGATAATAGCTAAAAATATCGTCCGTATACTATTATTTCAATTTCCGG   2590
JFZ    AAATATATTATATTACCTTCATTGATAATAGCTAAAAATATCGTCCGTATACTATTATTTCAATTTCCGG   2590
NY     AAATATATTATATTACCTTCATTGATAATAGCTAAAAATATCGTCCGTATACTATTATTTCAATTTCCGG   2590
XH     AAATATATTATATTACCTTCATTGATAATAGCTAAAAATATCGTCCGTATACTATTATTTCAATTTCCGG   2590
TY     AAATATATTATATTACCTTCATTGATAATAGCTAAAAATATCGTCCGTATACTATTATTTCAATTTCCGG   2590
YN-1   AAATATATTATATTACCTTCATTGATAATAGCTAAAAATATCGTCCGTATACTATTATTTCAATTTCCGG   2590

MLP    AATGGTATGAGGACTTAAAGGATTGGAATAGAGAAATGCATGTTAAATGTACCTATAACGGCGTTCAATT   2660
JFZ    AATGGTATGAGGACTTAAAGGATTGGAATAGAGAAATGCATGTTAAATGTACCTATAACGGCGTTCAATT   2660
NY     AATGGTATGAGGACTTAAAGGATTGGAATAGAGAAATGCATGTTAAATGTACCTATAACGGCGTTCAATT   2660
XH     AATGGTATGAGGACTTAAAGGATTGGAATAGAGAAATGCATGTTAAATGTACCTATAACGGCGTTCAATT   2660
TY     AATGGTATGAGGACTTAAAGGATTGGAATAGAGAAATGCATGTTAAATGTACCTATAACGGCGTTCAATT   2660
YN-1   AATGGTATGAGGACTTAAAGGATTGGAATAGAGAAATGCATGTTAAATGTACCTATAACGGCGTTCAATT   2660

MLP    ATCAGAAAAAGAATTTCCAAAAAACTGGTTAACAGACGGCATTCAGATCAAGATCCTATTTCCTTTTCGT   2730
JFZ    ATCAGAAAAAGAATTTCCAAAAAACTGGTTAACAGACGGCATTCAGATCAAGATCCTATTTCCTTTTCGT   2730
NY     ATCAGAAAAAGAATTTCCAAAAAACTGGTTAACAGACGGCATTCAGATCAAGATCCTATTTCCTTTTCGT   2730
XH     ATCAGAAAAAGAATTTCCAAAAAACTGGTTAACAGACGGCATTCAGATCAAGATCCTATTTCCTTTTCGT   2730
TY     ATCAGAAAAAGAATTTCCAAAAAACTGGTTAACAGACGGCATTCAGATCAAGATCCTATTTCCTTTTCGT   2730
YN-1   ATCAGAAAAAGAATTTCCAAAAAACTGGTTAACAGACGGCATTCAGATCAAGATCCTATTTCCTTTTCGT   2730

MLP    CTTAAACCTTGGCACAGATCTAAGTTACGAGCCCCTTATAATGATCCAATGAAAAAGCAAGGTCAAAAAA   2800
JFZ    CTTAAACCTTGGCACAGATCTAAGTTACGAGCCCCTTATAATGATCCAATGAAAAAGCAAGGTCAAAAAA   2800
NY     CTTAAACCTTGGCACAGATCTAAGTTACGAGCCTCTTATAACGATCCAATGAAAAAGCAAGGTCAAAAAA   2800
XH     CTTAAACCTTGGCACAGATCTAAGTTACGAGCCCCTTATAATGATCCAATGAAAAAGCAAGGTCAAAAAA   2800
TY     CTTAAACCTTGGCACAGATCTAAGTTACGAGCCCCTTATAATGATCCAATGAAAAAGCAAGGTCAAAAAA   2800
YN-1   CTTAAACCTTGGCACAGATCTAAGTTACGAGCCCCTTATAATGATCCAATGAAAAAGCAAGGTCAAAAAA   2800

MLP    ATGATTTTTGTTTTTTAACAATTTTTGGAATGGAAGCTGAACTACCTTTTGGTTCTCCCAGAAAAAAACT   2870
JFZ    ATGATTTTTGTTTTTTAACAATTTTTGGAATGGAAGCTGAACTACCTTTTGGTTCTCCCAGAAAAAAACT   2870
NY     ATGATTTTTGTTTTTTAACAATTTTTGGAATGGAAGCTGAACTACCTTTTGGTTCTCCCAGAAAAAAACT   2870
XH     ATGATTTTTGTTTTTTAACAATTTTTGGAATGGAAGCTGAACTACCTTTTGGTTCTCCCAGAAAAAAACT   2870
```

```
TY      ATGATTTTTGTTTTTTAACAATTTTTGGAATGGAAGCTGAACTACCTTTTGGTTCTCCCAGAAAAAAACT  2870
YN-1    ATGATTTTTGTTTTTTAACAATTTTTGGAATGGAAGCTGAACTACCTTTTGGTTCTCCCAGAAAAAAACT  2870

MLP     TTCATTTTTTGAACCGATTTTAAAAGAACTCAAAAAAAAAATTAAAAAATTGCAAAAGAAATGTTTTATA  2940
JFZ     TTCATTTTTTGAACCGATTTTAAAAGAACTCAAAAAAAAAATAAAAAAATTGCAAAAGAAATGTTTTATA  2940
NY      TTCATTTTTTGAACCGATTTTAAAAGAACTCAAAAAAAAAATTAAAAAATTGCAAAAGAAATGTTTTATA  2940
XH      TTCATTTTTTGAACCGATTTTAAAAGAACCCAAAAAAAAAATTAAAAAATTGCAAAAGAAATGTTTTCTA  2940
TY      TTCATTTTTTGAACCGATTTTAAAAGAACTCAAAAAAAAAATTAAAAAATTGCAAAAGAAATGTTTTATA  2940
YN-1    TTCATTTTTTGAACCGATTTTAAAAGAACTCAAAAAAAAAATTAAAAAATTGCAAAAGAAATGTTTTCTA  2940

MLP     ATTCTAGGAATTTTTAAAGAACAAACAAAATTGTTTCTAAATGTCTCAAAAAAACCAAAAAAATGGATCA  3010
JFZ     ATTCTAGGAATTTTTAAAGAACAAACAAAATTGTTTCTAAATGTCTCAAAAAAACCAAAAAAATGGATCA  3010
NY      ATTCTAGGAATTTTTAAAGAACAAACAAAATTGTTTCTAAATGTCTCAAAAAAACCAAAAAAATGGATCA  3010
XH      ATTCTAGGAATTTTTAAAGAACAAACAAAATTGTTTCTAAATGTCTCAAAAAAACCAAAAAAATGGATCA  3010
TY      ATTCTAGGAATTTTTAAAGAACAAACAAAATTGTTTCTAAATGTCTCAAAAAAACCAAAAAAATGGATCA  3010
YN-1    ATTCTAGGAATTTTTAAAGAACAAACAAAATTGTTTCTAAATGTCTCAAAAAAACCAAAAAAATGGATCA  3010

MLP     TTAAAAACATTCTGTTTATAAAAGAAATAATAAAAGAGCTCTCAAAAATAAACCCAATTGTATTATTTGG  3080
JFZ     TTAAAAACATTCTGTTTATAAAAGAAATAATAAAAGAACTCTCAAAAATAAACCCAATTGTATTATTTGG  3080
NY      TTAAAAACATTCTGTTTATAAAAGAAATAATAAAAGAACTCTCAAAAATAAACCCAATTATATTATTTGG  3080
XH      TTAAAAAAATTCTGTTTATAAAAGAAATAATAAAAGAACTCTCAAAAATAAACCCAATTGTATTATTTGG  3080
TY      TTAAAAACATTCTGTTTATAAAAGAAATAATAAAAGAACTCTCAAAAATAAACCCAATTGTATTATTTGG  3080
YN-1    TTAAAAACATTCTGTTTATAAAAGAAATAATAAAAGAACTCTCAAAAATAAACCCAATTGTATTATTTGG  3080

MLP     ATTAAGAGAAATAGAAGTATATGAATTGAGTGAAATTAAAAAAGATTCAATAATCAGTAATCGGATGATT  3150
JFZ     ATTAAGAGAAATAGAAGTATATGAATTGAGTGAAATTAAAAAAGATTCAATAATCAGTAATCGGATGATT  3150
NY      ATTGAGAGAAATAGAAGTATATGAATTGAGTGAAATTAAAAAAGATTCAATAATCAGTAATCGGATGATT  3150
XH      ATTAAGAGAAATAGAAGTATATGAATTGAGTGAAATTAAAAAAGATTCAATAATCAGTAATCGGATGATT  3150
TY      ATTAAGAGAAATAGAAGTATATGAATTGAGTGAAATTAAAAAAGATTCAATAATCAGTAATCGGATGATT  3150
YN-1    ATTAAGAGAAATAGAAGTATATGAATTGAGTGAAATTAAAAAAGATTCAATAATCAGTAATCGGATGATT  3150

MLP     CAGGAATCGTCCATTCAAATTAGATCTCAGGATTGGACAAATTATTCATTAACAGAAAAAAAAAATGCAAG  3220
JFZ     CAGGAATCGTCCATTCAAATTAGATCTCAGGATTGGACAAATTATTCATTAACAGAAAAAAAAAATGCAAG  3220
NY      CAGGAATCGTCCATTCAAATTAGATCTCAGGATTGGACAAATTATTCATTAACAGAAAAAAAAAATGCAAG  3220
XH      CAGGAATCGTCCATTCAAATTAGATCTCAGGATTGGACAAATTATTCATTAACAGAAAAAAAAAATGCAAG  3220
TY      CAGGAATCGTCCATTCAAATTAGATCTCAGGATTGGACAAATTATTCATTAACAGAAAAAAAAAATGCAAG  3220
YN-1    CAGGAATCGTCCATTCAAATTAGATCTCAGGATTGGACAAATTATTCATTAACAGAAAAAAAAAATGCAAG  3220

MLP     ATCTGACTGATAGAATAAACACAATCATAAATCAAATAGAAAAAATTACAAAAGACAAGAAAAAGGGATT  3290
JFZ     ATCTGACTGATAGAATAAACACAATCATAAATCAAATAGAAAAAATTACAAAAGACAAGAAAAAGGGATT  3290
NY      ATCTGACTGATAGAATAAACACAATCATAAATCAAATAGAAAAAATTACAAAAGACAAGAAAAAGGGATT  3290
```

```
XH     ATCTGACTGATAGAATAAACACAATCATAAATCAAATAGAAAAAATTACAAAAGACAAGAAAAAGGGATT  3290
TY     ATCTGACTGATAGAATAAACACAATCATAAATCAAATAGAAAAAATTACAAAAGACAAGAAAAAGGGATT  3290
YN-1   ATCTGACTGATAGAATAAACACAATCATAAATCAAATAGAAAAAATTACAAAAGACAAGAAAAAGGGATT  3290

MLP    TATAAAAGACAAGAAAAAGGGATTTATAACTTCAGATAGAAATTTGAGTTCTAACAAAATAAGTTATGAT  3360
JFZ    TA------------------------TAACTTCAGATAGAAATTTGAGTTCTAACAAAATAAGTTATGAT  3336
NY     TA------------------------TAACTTCAGATAGAAATTTGAGTTCTAACAAAATAAGTTATGAT  3336
XH     TATAAAAGACAAGAAAAAGGGATTTATAACTTCAGATAGAAATTTGAGTTCTAACAAAATAAGTTATGAT  3360
TY     TAAAAAAGACAAGAAAAAGGGATTTATAACTTCAGATAGAAATTTGAGTTCTAACAAAATAAGTTATGAT  3360
YN-1   TATAAAAGACAAGAAAAAGGGATTTATAACTTCAGATAGAAATTTGAGTTCTAACAAAATAAGTTATGAT  3360

MLP    GATAAAAGATTGGAATCACAAAAAAATATTTGGCAGATATTAAAAAGAGGAACTGCTCGATTAATCCGTA  3430
JFZ    GATAAAAGATTGGAATCACAAAAAAATATTTGGCAGATATTAAAAAGAGGAACTGCTCGATTAATCCGTA  3406
NY     GATAAAAGATTGGAATCACAAAAAAATATTTGGCAGATATTAAAAAGAAGAACTGCTCGATTAATCCGTA  3406
XH     GATAAAAGATTGGAATCACAAAAAAATATTTGGCAGATATTAAAAAGAGGAACTGCTCGATTAATCCGTA  3430
TY     GATAAAAGATTGGAATCACAAAAAAATATTTGGCAGATATTAAAAAGAGGAACTGCTCGATTAATCCGTA  3430
YN-1   GATAAAAGATTGGAATCACAAAAAAATATTTGGCAGATATTAAAAAGAGGAACTGCTCGATTAATCCGTA  3430

MLP    AATCCCATTATTTTATAATATTTTTCATTGAAAAGATATACATAGATATCTTTCTATCTATGATTAATAT  3500
JFZ    AATCCCATTATTTTATAATATTTTTCATTGAAAAGATATACATAGATATCTTTCTATCTATGATTAATAT  3476
NY     AATCCCATTATTTTATAATATTTTTCATTGAAAAGATATACATAGATATCTTTCTATCTATGATTAATAT  3476
XH     AATCCCATTATTTTATAATATTTTTCATTGAAAAGATATACATAGATATCTTTCTATCTATGATTAATAT  3500
TY     AATCCCATTATTTTATAATATTTTTCATTGAAAAGATATACATAGATATCTTTCTATCTATGATTAATAT  3500
YN-1   AATCCCATTATTTTATAATATTTTTCATTGAAAAGATATACATAGATATCTTTCTATCTATGATTAATAT  3500

MLP    TCCTAGTATCAATACACAACTTTTTCTTGAATCAACAAAAAAAATTATTAATAAAAACATTAACAGTAAT  3570
JFZ    TCCTAGTATCAATACACAACTTTTTCTTGAATCAACAAAAAAAATTATTAATAAAA------ACAGTAAT  3540
NY     TCCTAGTATCAATATACAACTTTTTCTTGAATCAACAAAAAAAATTATTAATAAAA------ACAGTAAT  3540
XH     TCCTAGTATCAATACACAACTTTTTCTTGAATCAACAAAAAAAATTATTAATAAAAACATTAACAGTAAT  3570
TY     TCCTAGTATCAATACACAACTTTTTCTTGAATCAACAAAAAAAATTATTAATAAAAACATTAACAGTAAT  3570
YN-1   TCCTAGTATCAATACACAACTTTTTCTTGAATCAACAAAAAAAATTATTAATAAAAACATTAACAGTAAT  3570

MLP    GAAGCAAATCAAGAAAGAATTAATAAAACAAATCAAAGTTTAATTAAATTTATTTCGATTATAAAAGAGT  3640
JFZ    GAAGCAAATCAAGAAAGAATTAATAAAACAAATCAAAGTTTAATTAAATTTATTTCGATTATAAAAGAGT  3610
NY     GAAGCAAATCAAGAAAGAATTAATAAAACAAATCAAAGTTTAATTAAATTTATTTCGATTATAAAAGAGT  3610
XH     GAAGCAAATCAAGAAAGAATTAATAAAACAAATCAAAGTTTAATTAAATTTATTTCGATTATAAAAGAGT  3640
TY     GAAGCAAATCAAGAAAGAATTAATAAAACAAATCAAAGTTTAATTAAATTTATTTCGATTATAAAAGAGT  3640
YN-1   GAAGCAAATCAAGAAAGAATTAATAAAACAAATCAAAGTTTAATTAAATTTATTTGGATTATAAAAGAGT  3640

MLP    CACTTTCTAATACTAATATTAGTCTTAGTCAAAAAAATTCAAAGACTTTTTTTGACTTATCTTACTTTTC  3710
JFZ    CACTTTCTAATACTAATATTAGTCTTAGTCAAAAAAATTCAAAGACTTTTTTTGACTTATCTTACTTTTC  3680
```

```
NY     CACTTTCTAATACTAATATTAGTCTTAGTCAAAAAAATTCAAAGACTTTTTTTGACTTATCTTACTTTTC  3680
XH     CACTTTCTAATACTAATATTAGTCTTAGTCAAAAAAATTCAAAGACTTTTTTTGACTTATCTTACTTTTC  3710
TY     CACTTTCTAATACTAATATTAGTCTTAGTCAAAAAAATTCAAAGACTTTTTTTGACTTATCTTACTTTTC  3710
YN-1   CACTTTCTAATACTAATATTAGTCTTAGTCAAAAAAATTCAAAGACTTTTTTTGACTTATCTTACTTTTC  3710

MLP    ACAAGCATATGTATTTTACAAATTATCACAAACCCAACTTATTAAGTTAGAGAAATTGAAATCCGTATTT  3780
JFZ    ACAAGCATATGTATTTTACAAATTATCACAAACCCAACTTATTAAGTTAGAGAAATTGAAATCCGTATTT  3750
NY     ACAAGCATATGTATTTTACAAATTATCACAAACCCAACTTATTAAGTTAGAGAAATTGAAATCCGTATTT  3750
XH     ACAAGCATATGTATTTTACAAATTATCACAAACCCAACTTATTAAGTTAGAGAAATTGAAATCCGTATTT  3780
TY     ACAAGCATATGTATTTTACAAATTATCACAAACCCAACTTATTAAGTTAGAGAAATTGAAATCCGTATTT  3780
YN-1   ACAAGCATATGTATTTTACAAATTATCACAAACCCAACTTATTAAGTTAGAGAAATTGAAATCCGTATTT  3780

MLP    CAATATAATGGAACCCCCCTTTTTCTTAAGAATGAAATAAAGGATTTTTTTGTTATAAGACAAGAACTAT  3850
JFZ    CAATATAATGGAACCTCCCTTTTTCTTAAGAATGAAATAAAGGATTTTTTTGTTATAAGACAAGAACTAT  3820
NY     CAATATAATGGAACCCCCCCTTTTCTTAAGAATGAAATAAAGGATTTTTTTGTTGTAAGACAAGAACTAT  3820
XH     CAATATAATGGAACCCCCCTTTTTCTTAAGAATGAAATAAAGGATTTTTTTGTTATAAGACAAGAACTAT  3850
TY     CAATATAATGGAACCCCCCTTTTTCTTAAGAATGAAATAAAGGATTTTTTTGTTATAAGACAAGAACTAT  3850
YN-1   CAATATAATGGAACCCCCCTTTTTCTTAAGAATGAAATAAAGGATTTTTTTGTTATAAGACAAGAACTAT  3850

MLP    TTCATTCCGAATTAAGACCTAAGAATCTTCGCAATTCTGGAATGAATCAATGGACAAATTGGTTAAGGAG  3920
JFZ    TTCATTCCGAATTAAGACCTAAGAATCTTCGCAATTCTGGAATGAATCAATGGACAAATTGGTTAAGGAG  3890
NY     TTCATTCCGAATTAAGACCTAAGAATCTTCGCAATTCTGGAATGAATCAATGGACAAATTGGTTAAGGAG  3890
XH     TTCATTCCGAATTAAGACCTAAGAATCTTCGCAATTCTGGAATGAATCAATGGACAAATTGGTTAAGGAG  3920
TY     TTCATTCCGAATTAAGACCTAAGAATCTTCGCAATTCTGGAATGAATCAATGGACAAATTGGTTAAGGAG  3920
YN-1   TTCATTCCGAATTAAGACCTAAGAATCTTCGCAATTCTGGAATGAATCAATGGACAAATTGGTTAAGGAG  3920

MLP    TCATTATCAATATCAATGTGATGTATCTCAAATTAAATGGTCTAGAGTAGTACCACAAAAATGGCGAAAT  3990
JFZ    TCATTATCAATATCAATGTGATGTATCTCAAATTAAATGGTCTAGAGTAGTACCACAAAAATGGCGAAAT  3960
NY     TCATTATCAAT------GTGATGTATCTCAAATTAAATGGTCTAGAGTAGTACCACAAAAATGGCGAAAT  3954
XH     TCATTATCAATATCAATGTGATGTATCTCAAATTAAATGGTCTAGAGTAGTACCACAAAAATGGCGAAAT  3990
TY     TCATTATCAATATCAATGTGATGTATCTCAAATTAAATGGTCTAGAGTAGTACCACAAAAATGGCGAAAT  3990
YN-1   TCATTATCAATATCAATGTGATGTATCTCAAATTAAATGGTCTAGAGTAGTACCACAAAAATGGCGAAAT  3990

MLP    ATATTGAACTGTATAGCTCAAAATAAAGATTTATATAAAGATTTGTGTGATTTATATGAAAAAGATGAAT  4060
JFZ    ATATTGAACTGTATAGCTCAAAATAAAGATTTATATAAAGATTTGTGTGATTTATATGAAAAAGATGAAT  4030
NY     ATATTGAACTGTATAGCTCAAAATAAAGATTTATATAAAGATTTGTGTGATTTATATGAAAAAGATGAAT  4024
XH     ATATTGAACTGTATAGCTCAAAATAAAGATTTATATAAAGATTTGTGTGATTTATATGAAAAAGATGAAT  4060
TY     ATATTGAACTGTATAGCTCAAAATAAAGATTTATATAAAGATTTGTGTGATTTATATGAAAAAGATGAAT  4060
YN-1   ATATTGAACTGTATAGCTCAAAATAAAGATTTATATAAAGATTTGTGTGATTTATATGAAAAAGATGAAT  4060

MLP    TAATTCACTACGAAAAAAAAACAAAAATTGAAACAGATTTATTATTGAATCAAAAGGATAATTTTAAAAA  4130
```

```
JFZ    TAATTCACTACGAAAAAAAAACAAAAATTGAAACAGATTTATTATTGAATCAAAAGGATAATTTTAAAAA  4100
NY     TAATTCACTACGAAAAAAAAACAAAAATTGAAACAGATTTATTATTGAATCAAAAGGATAATTTTAAAAA  4094
XH     TAATTCACTACGAAAAAAAAACAAAAATTGAAACAGATTTATTATTGAATCAAAAGGATAATTTTAAAAA  4130
TY     TAATTCACTACGAAAAAAAAACAAAAATTGAAACAGATTTATTATTGAATCAAAAGGATAATTTTAAAAA  4130
YN-1   TAATTCACTACGAAAAAAAAACAAAAATTGAAACAGATTTATTATTGAATCAAAAGGATAATTTTAAAAA  4130

MLP    ACAATATAGATATGATCTTTTAGCATATAAATCTATAAATTCTGAAACTAAGAAGTACTCTTCAATTTAT  4200
JFZ    ACAATATAGATATGATCTTTTAGCATATAAATCTATAAATTCTGAAACTAAGAAGTACTCTTCAATTTAT  4170
NY     ACAATATAGATATGATCTTTTAGCATATAAATCTATAAATTCTGAAACTAAGAAGTACTCTTCAATTTAT  4164
XH     ACAATATAGATATGATCTTTTAGCATATAAATCTATAAATTCTGAAACTAAGAAGTACTCTTCAATTTAT  4200
TY     ACAATATAGATATGATCTTTTAGCATATAAATCTATAAATTCTGAAACTAAGAAGTACTCTTCAATTTAT  4200
YN-1   ACAATATAGATATGATCTTTTAGCATATAAATCTATAAATTCTGAAACTAAGAAGTACTCTTCAATTTAT  4200

MLP    GGATCACCATTACAAGTAAAAACTAACCAAGATATTTTTTATAATTACAACACGCATAAACAAAAATCAT  4270
JFZ    GGATCACCATTACAAGTAAAAACTAACCAAGATATTTTTTATAATTACAACACGCATAAACAAAAATCAT  4240
NY     GGATCACCATTACAAGTAAAAACTAACCAAGATATTTTTTATAATTACAACACACATAAA AAAAATCAT  4234
XH     GGATCACCATTACAAGTAAAAACTAACCAAGATATTTTTTATAATTACAACACGCATAAACAAAAATCAT  4270
TY     GGATCACCATTACAAGTAAAAACTAACCAAGATATTTTTTATAATTACAACACGCATAAACAAAAATCAT  4270
YN-1   GGATCACCATTACAAGTAAAAACTAACCAAGATATTTTTTATAATTACAACACGCATAAACAAAAATCAT  4270

MLP    TTAATATGGTAGAAGATGATATTCGAGATATGGATAAAAATACGGATAGAAAATTTTTTGATTGGAGAAT  4340
JFZ    TTAATATGGTAGAAGATGATATTCGAGATATGGATAAAAATACGGATAGAAAATTTTTTGATTGGAGAAT  4310
NY     TTAATATGGTAGAAGATGATATTCGAGATATGGATAAAAATACGGATAGAAAATTTTTTGATTGGAGAAT  4304
XH     TTAATATGGTAGAAGATGATATTCGAGATATGGATAAAAATACGGATAGAAAATTTTTTGATTGGAGAAT  4340
TY     TTAATATGGTAGAAGATGATATTCGAGATATGGATAAAAATACGGATAGAAAATTTTTTGATTGGAGAAT  4340
YN-1   TTAATATGGTAGAAGATGATATTCGAGATATGGATAAAAATACGGATAGAAAATTTTTTGATTGGAGAAT  4340

MLP    TCTCGATTTTTGTCTTAAAACGAAGGTCGATATTGAGGCCTGGATCAATATTGATACTGACACTAACAGT  4410
JFZ    TCTCGATTTTTGTCTTAAAACGAAGGTCGATATTGAGGCCTGGATCAATATTGATACTGACACTAACAGT  4380
NY     TCTCGATTTTTGTCTTAAAACGAAGGTCGATATTGAGGCCTGGATCAATATTGATACTGACACTAACAGT  4374
XH     TCTCGATTTTTGTCTTAAAACGAAGGTCGATATTGAGGCCTGGATCAATATTGATACTGACACTAACAGT  4410
TY     TCTCGATTTTTGTCTTAAAACGAAGGTCGATATTGAGGCCTGGATCAATATTGATACTGACACTAACAGT  4410
YN-1   TCTCGATTTTTGTCTTAAAACGAAGGTCGATATTGAGGCCTGGATCAATATTGATACTGACACTAACAGT  4410

MLP    AATAAATATACTAAGACTAGGGTTAATAAGTATCAAATAATTGATAAAATCAATAATAAGGGTCTTTTTT  4480
JFZ    AATAAATATACTAAGACTAGGGTTAATAAGTATCAAATAATTGATAAAATCAATAATAAGGGTCTTTTTT  4450
NY     AATAAATATACTAAGACTGGGGTTAATAAGTATCAAATAATTGATAAAATCAATAATAAGGGTCTTTTTT  4444
XH     AATAAATATACTAAGACTAGGGTTAATAAGTATCAAATAATTGATAAAATCAATAATAAGGGTCTTTTTT  4480
TY     AATAAATATACTAAGACTAGGGTTAATAAGTATCAAATAATTGATAAAATCAATAATAAGGGTCTTTTTT  4480
YN-1   AATAAATATACTAAGACTAGGGTTAATAAGTATCAAATAATTGATAAAATCAATAATAAGGGTCTTTTTT  4480
```

```
MLP   ATCTCACACTTCAGCAAGATCAAAAAATCAACCTATCCAATCAAAAACCCCTTTTGGATTGGATGGGAAT  4550
JFZ   ATCTCACACTTCAGCAAGATCAAAAAATCAACCTATCCAATCAAAAACCCCTTTTGGATTGGATGGGAAT  4520
NY    ATCTCACACTTCAGCAAGATCAAAAAATCAACCTATCCAATCAAAAACCCCTTTTGGATTGGATGGGAAT  4514
XH    ATCTCACACTTCAGCAAGATCAAAAAATCAACCTATCCAATCAAAAACCCCTTTTGGATTGGATGGGAAT  4550
TY    ATCTCACACTTCAGCAAGATCAAAAAATCAACCTATCCAATCAAAAACCCCTTTTGGATTGGATGGGAAT  4550
YN-1  ATCTCACACTTCAGCAAGATCAAAAAATCAACCTATCCAATCAAAAACCCCTTTTGGATTGGATGGGAAT  4550

MLP   GAATGAAGAAATACTAAGTCGTCCCATATCAAATCTGGAACTTTGGTTCTTGCCAGAATTTGTGAGACTT  4620
JFZ   GAATGAAGAAATACTAAGTCGTCCCATATCAAATCTGGAACTTTGGTTCTTGCCAGAATTTGTGAGACTT  4590
NY    GAATGAAGAAATACTAAGTCGTCCCATATCAAATCTGGAACTTTGGTTCTTGCCAGAATTTTTGAGACTT  4584
XH    GAATGAAGAAATACTAAGTCGTCCCATATCAAATCTGGAACTTTGGTTCTTGCCAGAATTTGTGAGACTT  4620
TY    GAATGAAGAAATACTAAGTCGTCCCATATCAAATCTGGAACTTTGGTTCTTGCCAGAATTTGTGAGACTT  4620
YN-1  GAATGAAGAAATACTAAGTCGTCCCATATCAAATCTGGAACTTTGGTTCTTGCCAGAATTTGTGAGACTT  4620

MLP   TATAATACGTATAAAATGAAACCGTGGGTTATACCAATTAAATTACTACTTTTTAATTCTAATATAAAAA  4690
JFZ   TATAATACGTATAAAATGAAACCGTGGGTTATACCAATTAAATTACTACTTTTTAATTCTAATATAAAAA  4660
NY    TATAATACGTATAAAATGAAACCGTGGGTTATACCAATTAAATTACTACTTTTTAATTCTAATATAAAAA  4654
XH    TATAATACGTATAAAATGAAACCGTGGGTTATACCAATTAAATTACTACTTTTTAATTCTAATATAAAAA  4690
TY    TATAATACGTATAAAATGAAACCGTGGGTTATACCAATTAAATTACTACTTTTTAATTCTAATATAAAAA  4690
YN-1  TATAATACGTATAAAATGAAACCGTGGGTTATACCAATTAAATTACTACTTTTTAATTCTAATATAAAAA  4690

MLP   AAAATGCTAGTGAAAACAAAAGCATCACTGGAAATAAAAAAAGAGATCCTTTTATATCAATATCGTCGAA  4760
JFZ   AAAATGCTAGTGAAAACAAAAGCATCACTGGAAATAAAAAAAGAGATCCTTTTATATCAATATCGTCGAA  4730
NY    AAAATGCTAGTGAAAACAAAAGCATCACTGGAAATAAAAAAAGAGATCCTTTTATATCAATATCGTCGAA  4724
XH    AAAATGCTAGTGAAAACAAAAGCATCACTGGAAATAAAAAAAGAGATCCTTTTATATCAATATCGTCGAA  4760
TY    AAAATGCTAGTGAAAACAAAAGCATCACTGGAAATAAAAAAAGAGATCCTTTTATATCAATATCGTCGAA  4760
YN-1  AAAATGCTAGTGAAAACAAAAGCATCACTGGAAATAAAAAAAGAGATCCTTTTATATCAATATCGTCGAA  4760

MLP   TGAAAAAAAATCTCTTGAATTAGAAAACCGAAATCAAGGAGAAAAAGAATCCACGGGCCAAGCAGATCTT  4830
JFZ   TGAAAAAAAATCTCTTGAATTAGAAAACCGAAATCAAGGAGAAAAAGAATCCACGGGCCAAGCAGATCTT  4800
NY    TGAAAAAAAATCTCTTGAATTAGAAAACCGAAATCAAGGAGAAAAAGAATCCACGGGCCAAGCAGATCTT  4794
XH    TGAAAAAAAATCTCTTGAATTAGAAAACCGAAATCAAGGAGAAAAAGAATCCACGGGCCAAGCAGATCTT  4830
TY    TGAAAAAAAATCTCTTGAATTAGAAAACCGAAATCAAGGAGAAAAAGAATCCACGGGCCAAGCAGATCTT  4830
YN-1  TGAAAAAAAATCTCTTGAATTAGAAAACCGAAATCAAGGAGAAAAAGAATCCACGGGCCAAGCAGATCTT  4830

MLP   AAATCAGCTCTTTCAAACCAAGAAAAAGATGTTGAAGAAGATTATACGGGATCGGACATGAAAAAACATA  4900
JFZ   AAATCAGCTCTTTCAAACCAAGAAAAAGATGTTGAAGAAGATTATACGGGATCGGACATGAAAAAACATA  4870
NY    AAATCAGCTCTTTCAAACCAAGAAAAAGATGTTGAAGAAGATTATACGGGATCGGACATGAAAAAACATA  4864
XH    AAATCAGCTCTTTCAAACCAAGAAAAAGATGTTGAAGAAGATTATACGGGATCGGACATGAAAAAACATA  4900
TY    AAATCAGCTCTTTCAAACCAAGAAAAAGATGTTGAAGAAGATTATACGGGATCGGACATGAAAAAACATA  4900
YN-1  AAATCAGCTCTTTCAAACCAAGAAAAAGATGTTGAAGAAGATTATACGGGATCGGACATGAAAAAACATA  4900
```

```
MLP   GAAATAAAAAGCAATACAAGATCAATACAAAAGCGGAGTTTGATTTCTTCCTAAAAAGGTATTTGTATTT  4970
JFZ   GAAATAAAAAGCAATACAAGATCAATACAAAAGCGGAGTTTGATTTCTTCCTAAAAAGGTATTTGTATTT  4940
NY    GAAATAAAAAGCAATACAAGATCAATACAAAAGCGGAGTTTGATTTCTTCCTAAAAAGGTATTTGTATTT  4934
XH    GAAATAAAAAGCAATACAAGATCAATACAAAAGCGGAGTTTGATTTCTTCCTAAAAAGGTATTTGTATTT  4970
TY    GAAATAAAAAGCAATACAAGATCAATACAAAAGCGGAGTTTGATTTCTTCCTAAAAAGGTATTTGTATTT  4970
YN-1  GAAATAAAAAGCAATACAAGATCAATACAAAAGCGGAGTTTGATTTCTTCCTAAAAAGGTATTTGTATTT  4970

MLP   TCAATTGAGATGGGATGATTCTTTAAATAAAAAAATAATCAATAACATCAACGTATATTGCCTCCTGCTT  5040
JFZ   TCAATTGAGATGGGATGATTCTTTAAATAAAAAAATAATCAATAACATCAACGTATATTGCCTCCTGCTT  5010
NY    TCAATTGAGATGGGATGATTCTTTAAATAAAAAAATAATCAATAACATCAACGTATATTGTCTCCTGCTT  5004
XH    TCAATTGAGATGGGATGATTCTTTAAATAAAAAAATAATCAATAACATCAACGTATATTGCCTCCTGCTT  5040
TY    TCAATTGAGATGGGATGATTCTTTAAATAAAAAAATAATCAATAACATCAACGTATATTGCCTCCTGCTT  5040
YN-1  TCAATTGAGATGGGATGATTCTTTAAATAAAAAAATAATCAATAACATCAACGTATATTGCCTCCTGCTT  5040

MLP   AGACTGATAAATCCAAGAGAAATTACTATATCCTCTATTCAAAGGGGCGAAATGAGTCTGGATATTTTGA  5110
JFZ   AGACTGATAAATCCAAGAGAAATTACTATATCCTCTATTCAAAGGGGCGAAATGAGTCTGGATATTTTGA  5080
NY    AGACTGATAAATCCAAGAGAAATTACTATATCCTCTATTCAAAGGGGCGAAATGAGTCTGGATATTTTGA  5074
XH    AGACTGATAAATCCAAGAGAAATTACTATATCCTCTATTCAAAGGGGCGAAATGAGTCTGGATATTTTGA  5110
TY    AGACTGATAAATCCAAGAGAAATTACTATATCCTCTATTCAAAGGGGCGAAATGAGTCTGGATATTTTGA  5110
YN-1  AGACTGATAAATCCAAGAGAAATTACTATATCCTCTATTCAAAGGGGCGAAATGAGTCTGGATATTTTGA  5110

MLP   CGATTCAGAAAGATGTAACTCTTACAGAATTTATTAAAAGGGGATTTTTGATTATTGAACCAATTCGTCT  5180
JFZ   CGATTCAGAAAGATGTAACTCTTACAGAATTTATTAAAAGGGGATTTTTGATTATTGAACCAATTCGTCT  5150
NY    CGATTCAGAAAGATGTAACTCTTACAGAATTTATTAAAAGGGGATTTTTGATTATTGAACCAATTCGTCT  5144
XH    CGATTCAGAAAGATGTAACTCTTACAGAATTTATTAAAAGGGGATTTTTGATTATTGAACCAATTCGTCT  5180
TY    CGATTCAGAAAGATGTAACTCTTACAGAATTTATTAAAAGGGGATTTTTGATTATTGAACCAATTCGTCT  5180
YN-1  CGATTCAGAAAGATGTAACTCTTACAGAATTTATTAAAAGGGGATTTTTGATTATTGAACCAATTCGTCT  5180

MLP   AGCTATAAAAAATGATGGAAAATTTATTATGTATCAAACCCTCGGTATTTCATTAGTTCATAAAAGTAAA  5250
JFZ   AGCTATAAAAAATGATGGAAAATTTATTATGTATCAAACCCTCGGTATTTCATTAGTTCATAAAAGTAAA  5220
NY    AGCTATAAAAAATGATGGAAAATTTATTATGTATCAAACCCTCGGTATTTCATTAGTTCATAAAAGTAAA  5214
XH    AGCTATAAAAAATGATGGAAAATTTATTATGTATCAAACCCTCGGTATTTCATTAGTTCATAAAAGTAAA  5250
TY    AGCTATAAAAAATGATGGAAAATTTATTATGTATCAAACCCTCGGTATTTCATTAGTTCATAAAAGTAAA  5250
YN-1  AGCTATAAAAAATGATGGAAAATTTATTATGTATCAAACCCTCGGTATTTCATTAGTTCATAAAAGTAAA  5250

MLP   CATCAAATAAATCAAAGATACCGAGAAAAAAAACATGTTGATAAGAATTCTGATGAAGTCATTACAAAAC  5320
JFZ   CATCAAATAAATCAAAGATACCGAGAAAAAAAACATGTTGATAAGAATTCTGATGAAGTCATTACAAAAC  5290
NY    CATCAAATAAATCAAAGATACCGAGAAAAAAAACATGTTGATAAGAATTCTGATGAAGTCATTACAAAAC  5284
XH    CATCAAATAAATCAAAGATACCGAGAAAAAAAACATGTTGATAAGAATTCTGATGAAGTCATTACAAAAC  5320
TY    CATCAAATAAATCAAAGATACCGAGAAAAAAAACATGTTGATAAGAATTCTGATGAAGTCATTACAAAAC  5320
```

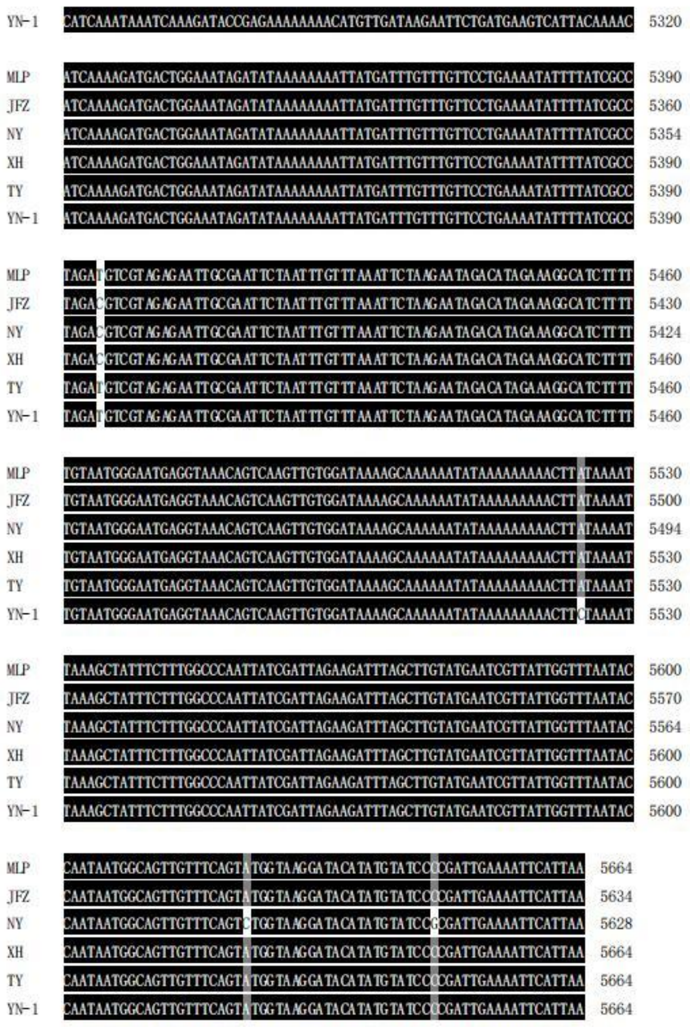

Fig. S1 Alignment of ycf1 genes of six different species of loquats.

Figure S2

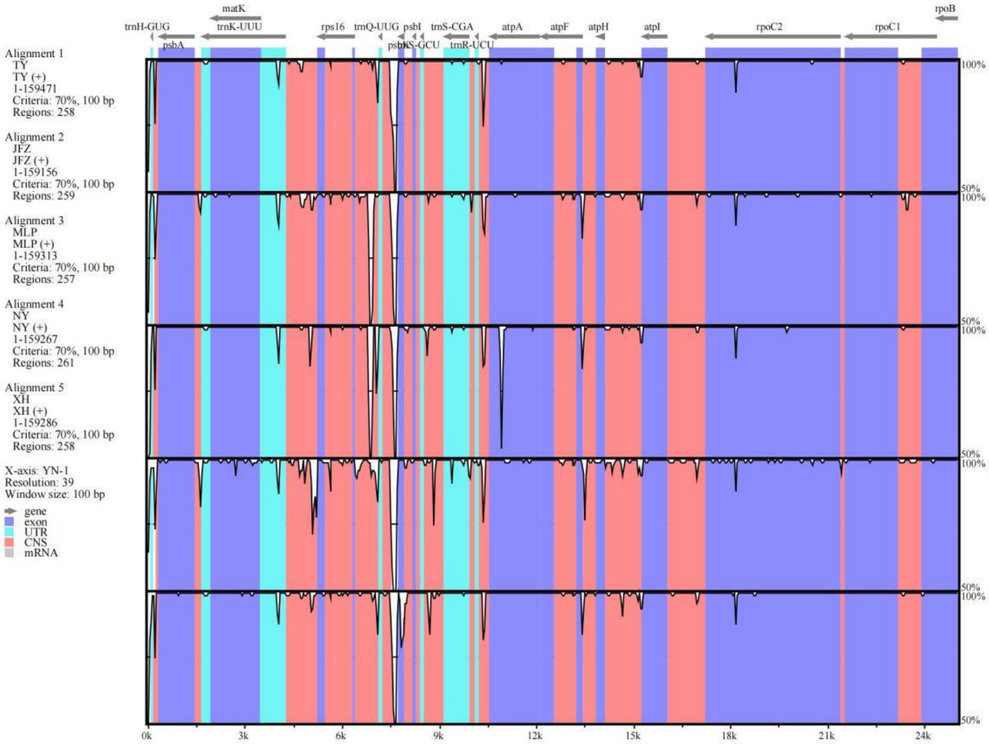

matK
trnH-GUG
psbA
trnK-UUU
rps16
trnQ-UUG
psbI
trnS-CGA
trnR-UCU
atpA
atpF
atpH
atpI
rpoC2
rpoC1
rpoB
Alignment 1
TY
TY (+)
1-159471
Criteria: 70%, 100 bp
Regions: 258
Alignment 2
JFZ
JFZ (+)
1-159156
Criteria: 70%, 100 bp
Regions: 259
Alignment 3
MLP
MLP (+)
1-159313
Criteria: 70%, 100 bp
Regions: 257
Alignment 4
NY
NY (+)
1-159267
Criteria: 70%, 100 bp
Regions: 261
Alignment 5
XH
XH (+)
1-159286
Criteria: 70%, 100 bp
Regions: 258
X-axis: YN-1
Resolution: 39
Window size: 100 bp
gene
exon
UTR
CNS
mRNA
100%
50%
0k
3k
6k
9k
12k
15k
18k
21k
24k

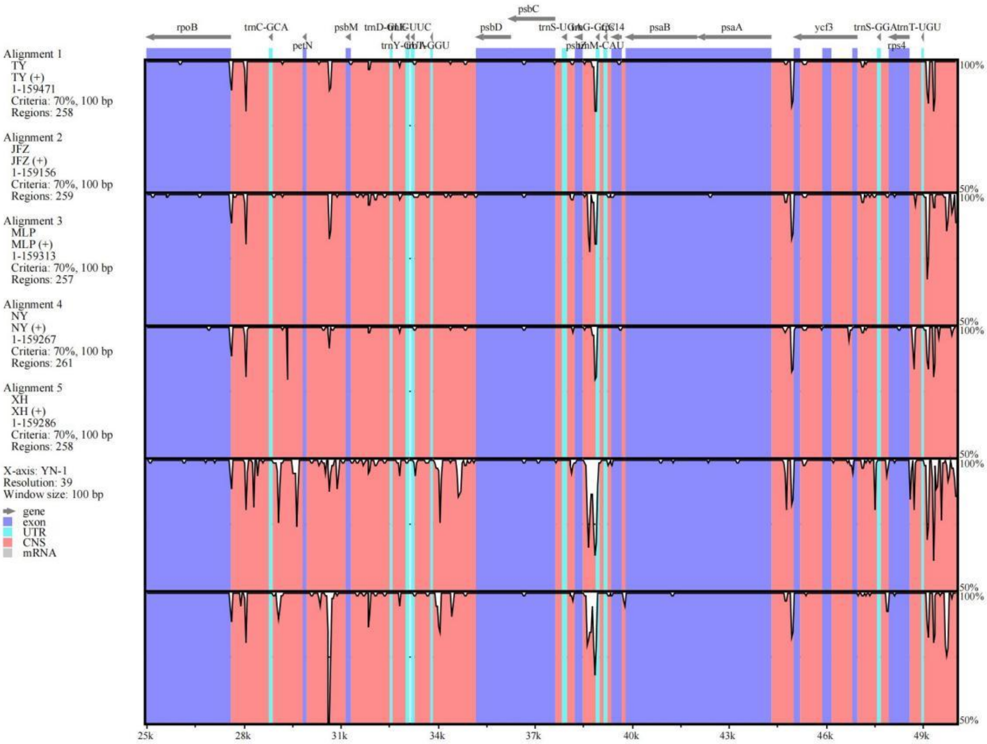

rpoB
trnC-GCA
petN
psbM
psbC
psbD
psaB
psaA
ycf3
rps4
Alignment 1
TY
TY (+)
1-159471
Criteria: 70%, 100 bp
Regions: 258
Alignment 2
JFZ
JFZ (+)
1-159156
Criteria: 70%, 100 bp
Regions: 259
Alignment 3
MLP
MLP (+)
1-159313
Criteria: 70%, 100 bp
Regions: 257
Alignment 4
NY
NY (+)
1-159267
Criteria: 70%, 100 bp
Regions: 261
Alignment 5
XH
XH (+)
1-159286
Criteria: 70%, 100 bp
Regions: 258
X-axis: YN-1
Resolution: 39
Window size: 100 bp
gene
exon
UTR
CNS
mRNA
100%
50%
25k
28k
31k
34k
37k
40k
43k
46k
49k

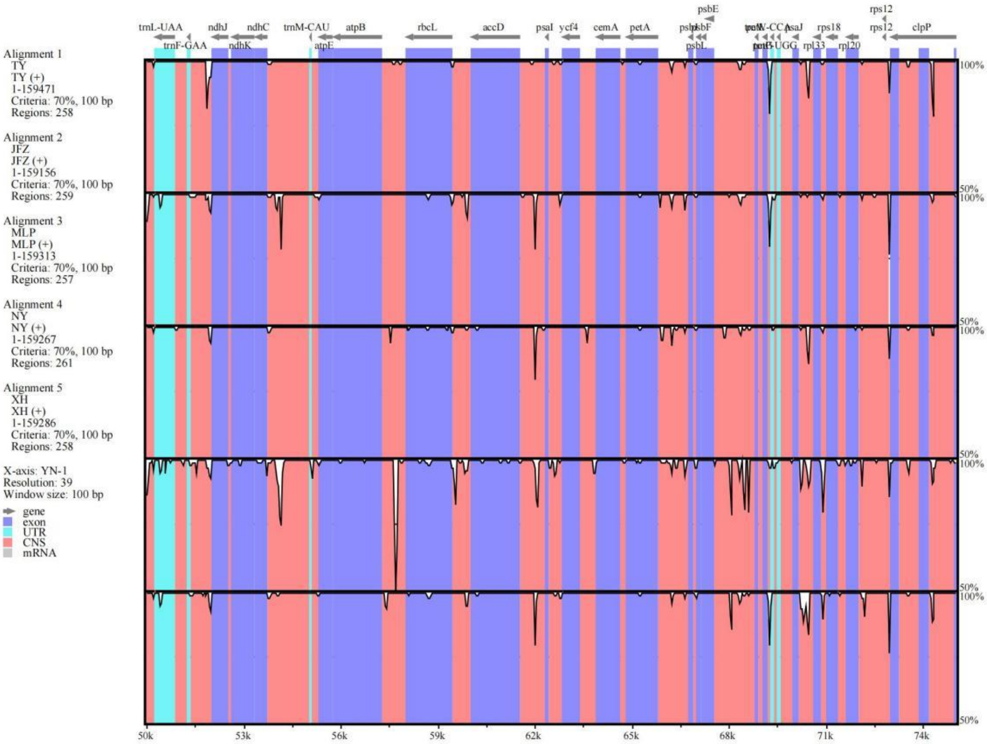
Alignment 1
TY
TY (+)
1-159471
Criteria: 70%, 100 bp
Regions: 258
Alignment 2
JFZ
JFZ (+)
1-159156
Criteria: 70%, 100 bp
Regions: 259
Alignment 3
MLP
MLP (+)
1-159313
Criteria: 70%, 100 bp
Regions: 257
Alignment 4
NY
NY (+)
1-159267
Criteria: 70%, 100 bp
Regions: 261
Alignment 5
XH
XH (+)
1-159286
Criteria: 70%, 100 bp
Regions: 258
X-axis: YN-1
Resolution: 39
Window size: 100 bp
gene
exon
UTR
CNS
mRNA
trnL-UAA
trnF-GAA
ndhJ
ndhK
ndhC
trnM-CAU
atpE
atpB
rbcL
accD
psaI
ycf4
cemA
petA
psbE
psbF
psbL
rps12
rpl33
rps18
rpl20
clpP
100%
50%
50k
53k
56k
59k
62k
65k
68k
71k
74k

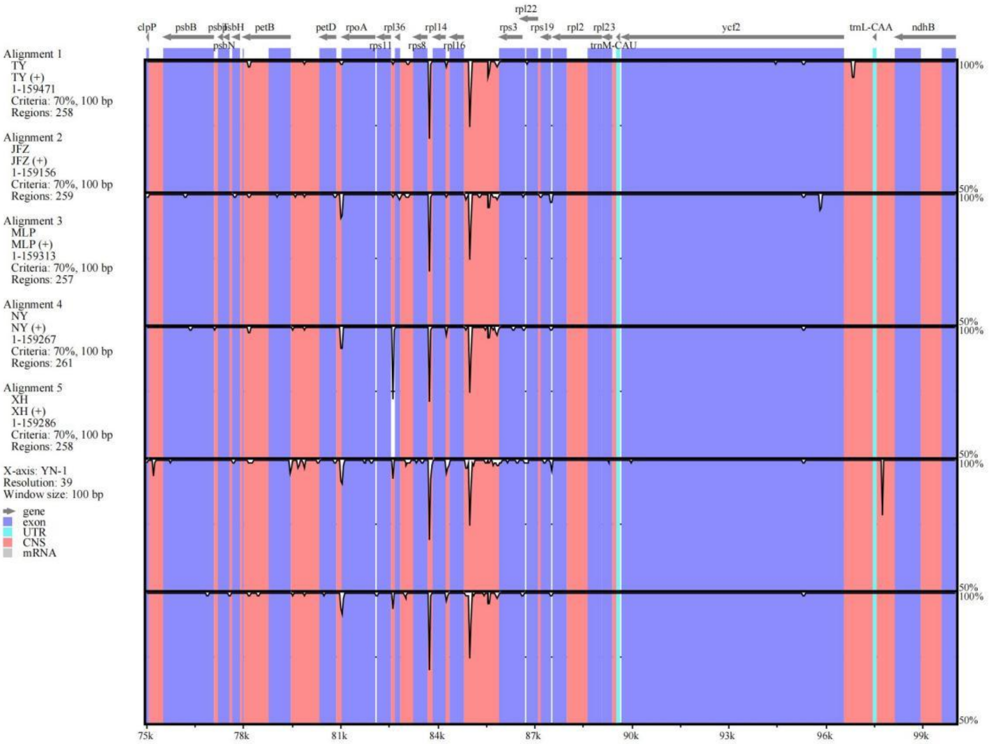
rpl22
clpP
psbB
psbT
psbH
psbN
petB
petD
rpoA
rpl36
rps11
rps8
rpl14
rpl16
rps3
rps19
rpl2
rpl23
trnM-CAU
ycf2
trnL-CAA
ndhB
Alignment 1
TY
TY (+)
1-159471
Criteria: 70%, 100 bp
Regions: 258
Alignment 2
JFZ
JFZ (+)
1-159156
Criteria: 70%, 100 bp
Regions: 259
Alignment 3
MLP
MLP (+)
1-159313
Criteria: 70%, 100 bp
Regions: 257
Alignment 4
NY
NY (+)
1-159267
Criteria: 70%, 100 bp
Regions: 261
Alignment 5
XH
XH (+)
1-159286
Criteria: 70%, 100 bp
Regions: 258
X-axis: YN-1
Resolution: 39
Window size: 100 bp
gene
exon
UTR
CNS
mRNA
100%
50%
75k
78k
81k
84k
87k
90k
93k
96k
99k

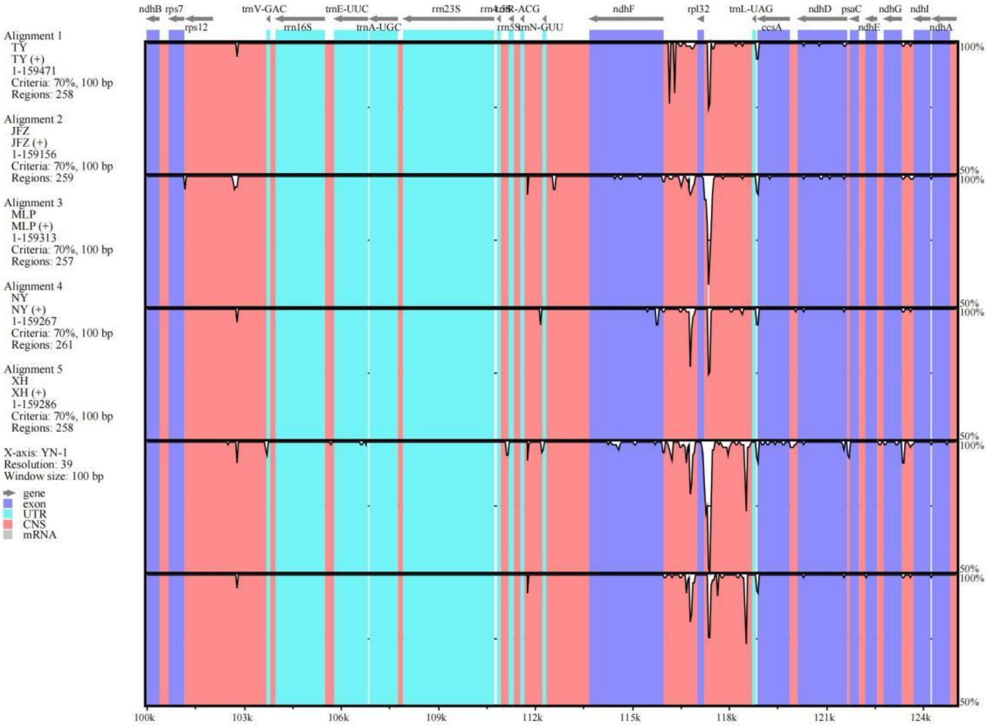

ndhB
rps7
rps12
trnV-GAC
rrn16S
trnE-UUC
trnA-UGC
rrn23S
rrn5S
trnN-GUU
ndhF
rpl32
trnL-UAG
ccsA
ndhD
psaC
ndhE
ndhG
ndhI
ndhA
Alignment 1
TY
TY (+)
1-159471
Criteria: 70%, 100 bp
Regions: 258
Alignment 2
JFZ
JFZ (+)
1-159156
Criteria: 70%, 100 bp
Regions: 259
Alignment 3
MLP
MLP (+)
1-159313
Criteria: 70%, 100 bp
Regions: 257
Alignment 4
NY
NY (+)
1-159267
Criteria: 70%, 100 bp
Regions: 261
Alignment 5
XH
XH (+)
1-159286
Criteria: 70%, 100 bp
Regions: 258
X-axis: YN-1
Resolution: 39
Window size: 100 bp
gene
exon
UTR
CNS
mRNA
100%
50%
100k
103k
106k
109k
112k
115k
118k
121k
124k

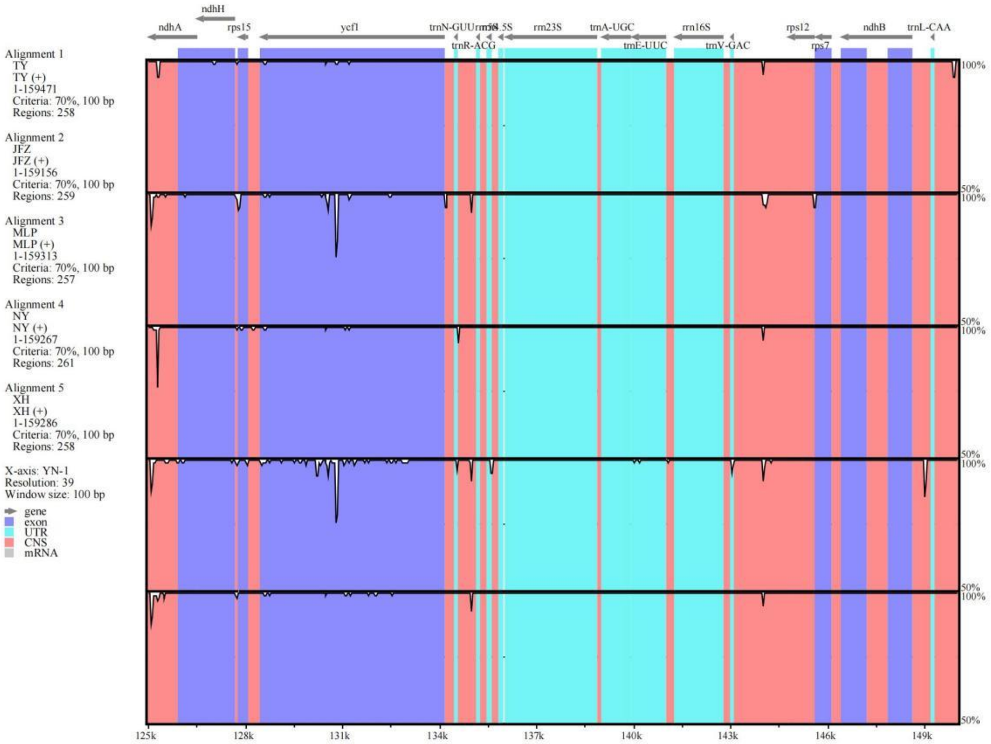

ndhH
ndhA
rps15
ycf1
trnN-GUU
rrn5
rrn4.5S
rrn23S
trnA-UGC
rrn16S
rps12
ndhB
trnL-CAA
trnR-ACG
trnE-UUC
trnV-GAC
rps7
Alignment 1
TY
TY (+)
1-159471
Criteria: 70%, 100 bp
Regions: 258
Alignment 2
JFZ
JFZ (+)
1-159156
Criteria: 70%, 100 bp
Regions: 259
Alignment 3
MLP
MLP (+)
1-159313
Criteria: 70%, 100 bp
Regions: 257
Alignment 4
NY
NY (+)
1-159267
Criteria: 70%, 100 bp
Regions: 261
Alignment 5
XH
XH (+)
1-159286
Criteria: 70%, 100 bp
Regions: 258
X-axis: YN-1
Resolution: 39
Window size: 100 bp
gene
exon
UTR
CNS
mRNA
100%
50%
125k
128k
131k
134k
137k
140k
143k
146k
149k

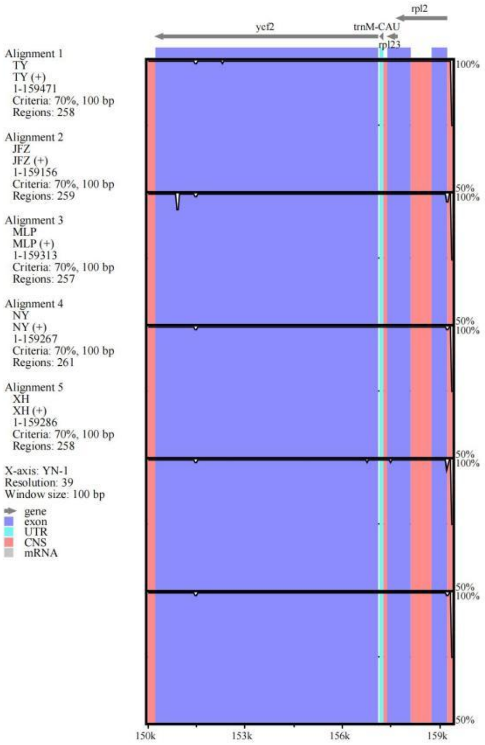

Fig S2 Visual alignment of six different species of loquats' chloroplast genome, "YN-1" chloroplast genome sequence as a reference.

Figure S3

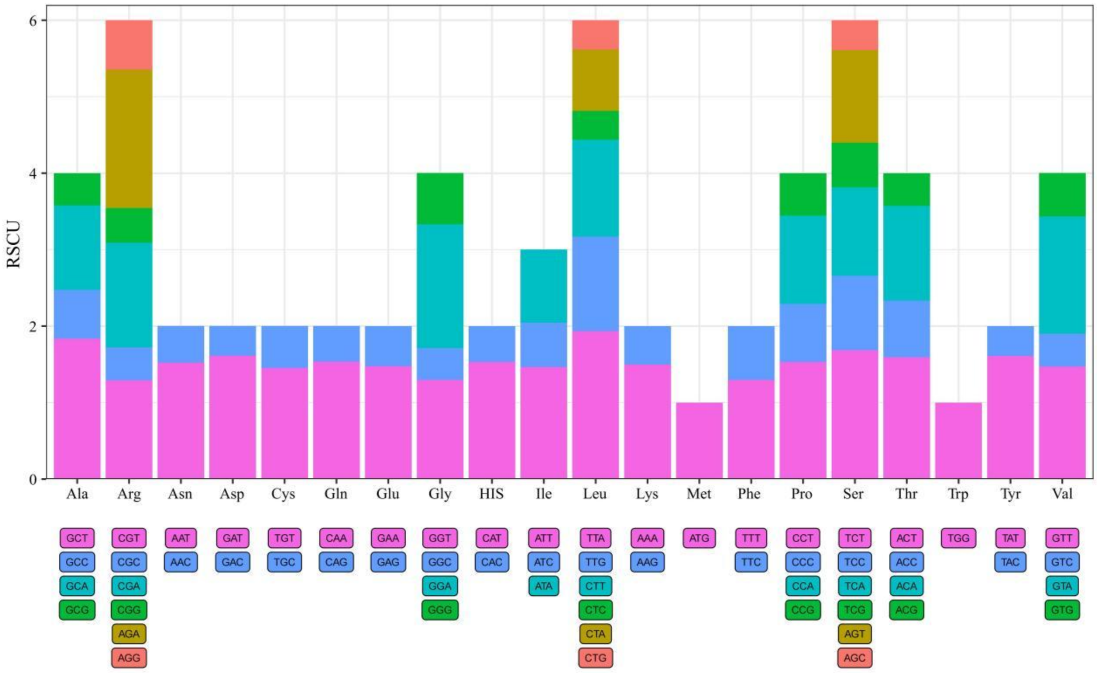

Fig S3 Relative Synonymous Codon Usage (RSCU) of wild loquat “TY”.
